# FaPOD27 functions in the metabolism of polyphenols in strawberry fruit (*Fragaria* sp.)

**DOI:** 10.3389/fpls.2014.00518

**Published:** 2014-10-09

**Authors:** Su-Ying Yeh, Fong-Chin Huang, Thomas Hoffmann, Mechthild Mayershofer, Wilfried Schwab

**Affiliations:** Biotechnology of Natural Products, WZW - TUM School of Life Sciences Weihenstephan, Technische Universität MünchenFreising, Germany

**Keywords:** strawberry, lignification, peroxidase, fruit firmness, monolignol genes

## Abstract

The strawberry (*Fragaria* × *ananassa*) is one of the most preferred fresh fruit worldwide, accumulates numerous flavonoids but has limited shelf life due to excessive tissue softening caused by cell wall degradation. Since lignin is one of the polymers that strengthen plant cell walls and might contribute to some extent to fruit firmness monolignol biosynthesis was studied in strawberry fruit. Cinnamoyl-CoA reductase (*CCR*), cinnamyl alcohol dehydrogenase (*CAD*), and a peroxidase (*POD27*) gene were strongly expressed in red, ripe fruit whereas a second *POD* gene was primarily expressed in green, immature fruit. Moreover, *FaPOD*27 transcripts were strongly and constitutively induced in fruits exposed to *Agrobacterium* infection. Gene expression levels and enzymatic activities of FaCCR and FaCAD were efficiently suppressed through RNAi in *FaCCR- and FaCAD*-silenced strawberries. Besides, significantly elevated *FaPOD* transcript levels were detected after agroinfiltration of pBI-*FaPOD* constructs in fruits. At the same time, levels of G-monomers were considerably reduced in *FaCCR*-silenced fruits whereas the proportion of both G- and S-monomers decisively decreased in *FaCAD*-silenced and pBI-*FaPOD* fruits. Development, firmness, and lignin level of the treated fruits were similar to pBI-intron control fruits, presumably attributed to increased expression levels of *FaPOD27* upon agroinfiltration. Additionally, enhanced firmness, accompanied with elevated lignin levels, was revealed in chalcone synthase-deficient fruits (CHS^−^), independent of down- or up-regulation of individual and combined *FaCCR*. *FaCAD*, and *FaPOD* genes by agroinfiltration, when compared to CHS^−^/pBI-intron control fruits. These approaches provide further insight into the genetic control of flavonoid and lignin synthesis in strawberries. The results suggest that *FaPOD27* is a key gene for lignin biosynthesis in strawberry fruit and thus to improving the firmness of strawberries.

## Introduction

The strawberry (*Fragaria* × *ananassa*) is a highly perishable fruit, with a short shelf life due to increased cell wall degradation during the late stages of ripening, which results in soft fruits (Lefever et al., 2004). Thus, fruit texture plays an important role as a quality marker for consumers and food processing companies since fruit softening renders the fruit palatable but facilitates pathogen infection and accelerates fruit postharvest decay. Consequently, fruit firmness is an important target for genetic engineering to improve the quality of strawberries and to prolong the shelf-life of fresh fruit in markets (Chapple and Carpita, 1998; Manning, 1998).

As fruit softening is associated with cell wall disassembly (Seymour and Gross, 1996) several studies aimed at slowing down this degradation process to improve the texture of strawberries. The pectate lyase gene is highly expressed during strawberry fruit ripening and is considered to play an important role in pectin decomposition (Dominguez-Puigjaner et al., 1997; Medina-Escobar et al., 1997). Transgenic strawberry fruits harboring antisense pectate lyase genes resulted in reduced pectate lyase activity, as well as increased fruit firmness (Jiménez-Bermúdez et al., 2002; Quesada et al., 2009).

To identify candidate genes which might be associated with textural differences in Fragaria accessions, gene expression levels were compared for a soft and a firm cultivar (Salentijn et al., 2003). Although putative cell wall related genes displayed differential expression, two genes of the lignin biosynthesis pathway namely cinnamoyl-CoA reductase (*CCR*) and cinnamyl alcohol dehydrogenase (*CAD*) showed the highest difference in expression level. The *CCR* gene showed higher expression levels in varieties that produce fruits with soft tissue, while *CAD* showed higher levels in the varieties with firm fruit tissue (Salentijn et al., 2003). Similarly, in loquat fruit (*Eriobotrya japonica* Lindl.), an increase in fruit firmness is associated with lignification of plant tissue and with activities of CAD and peroxidase (POD) enzymes that are involved in lignin biosynthesis (Boerjan et al., 2003; Cai et al., 2006). CCR (EC 1.2.1.44) is the first committed enzyme of the lignin branch biosynthetic pathway, and it mainly reduces cinnamoyl-CoA esters to yield corresponding aldehydes (Sarni et al., 1984). CCR has been considered to be a potential control point in regulating carbon flux toward lignin production (Lacombe et al., 1997). CAD (EC 1.1.1.195) catalyzes the conversion of cinnamyl aldehydes to their corresponding monolignols that are precursors for lignin polymerization in cell walls (Sarni et al., 1984) whereas POD (EC1.11.1.7) is responsible for the polymerization of monolignols *via* radical-radical coupling reactions (Almagro et al., 2009).

Lignin is derived from oxidative polymerization of three different monolignols (*p*-coumaryl, coniferyl and sinapyl alcohol) referred to as *p*-hydroxyphenyl (H), guaiacyl (G), and syringyl (S) lignin, respectively. However, due to the generally broad substrate tolerance of POD enzymes various substrates such as monolignols, ferulic acids, hydroxycinnamyl aldehydes, and acylated monolignols, can be incorporated into the branched lignin (Passardi et al., 2004b; Hayashi, 2006; Ralph et al., 2008; Weng and Chapple, 2010). PODs produce phenoxy radicals that form linkages (diphenolic) between cell wall polymers, causing cross-link formation and stiffening of the cell wall (Passardi et al., 2004a). Thus, lignin may functions as mechanical support for plant organs, in defense against pathogenic attacks, and in water transport in vascular plants (Dixon, 2001; Boerjan et al., 2003).

**Figure 1:**
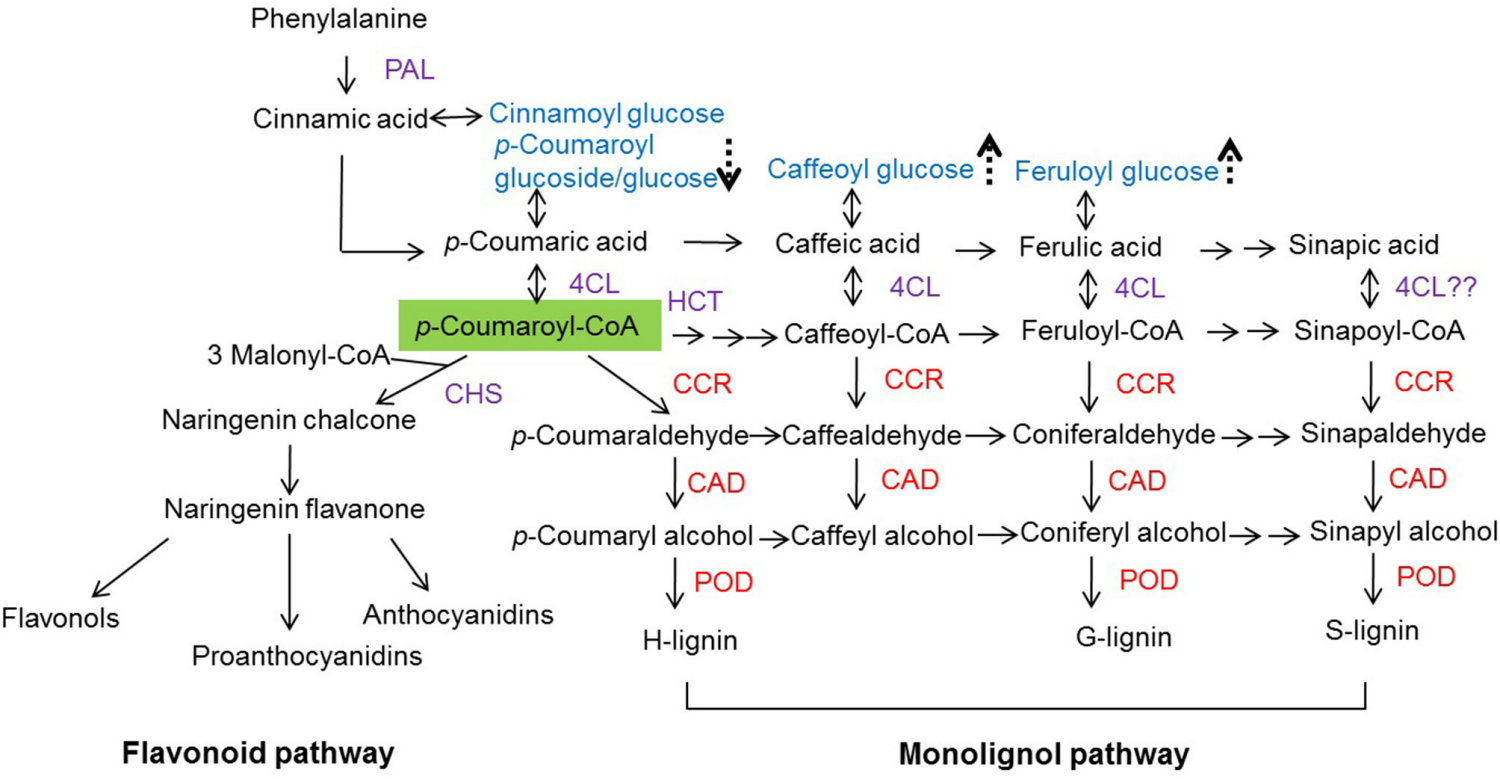
**Flavonoid and monolignol biosynthetic pathways**. PAL, phenylalanine ammonia lyase; 4CL, 4-coumaroyl-CoA ligase; CHS, chalcone synthase; HCT, hydroxycinnamoyl transferase; CCR, cinnamoyl-CoA reductase; CAD, cinnamyl alcohol dehydrogenase; POD, peroxidase. Arrow with dashed lines indicate compounds are up- (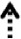) or down-regulated (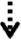) when fruits were agroinfiltrated pBI-intron (control constructs), down- or up-regulation of individual genes (*FaCCR*. *FaCAD*, or *FaPOD*) (Fig. S12). The green shade indicating *p*-coumaroyl-CoA is the common substrate of CHS, HCT, and CCR. Red *CCR*. *CAD*, and *POD* indicate down- and up-regulated genes.

Angiosperm lignin mainly contains both G and S units, and a low content of H units. A difference in lignin composition is reflected in the substrate specificity of enzymes or the regulated differential carbon flow into the synthesis of various lignin precursors in lignin biosynthesis. Indeed, lignin represents a carbon sink in higher plants. *p*-Coumaroyl-CoA is situated at the branching point of the metabolic routes leading to either flavonoid or monolignol biosynthetic pathways, as it is the common substrate of chalcone synthase (CHS), hydroxycinnamoyl transferase (HCT), and CCR enzymes (Figure 1; Boerjan et al., 2003; Besseau et al., 2007). When the carbon flow down the flavonoid pathway becomes limited, *p*-coumaroyl-CoA provides increased levels of monolignols (Lunkenbein et al., 2006).

The monomeric units in lignin are linked together through at least six different types of bonding, with the β-O-4 (β-aryl ether) group being the most frequent linkage (Boerjan et al., 2003; Weng and Chapple, 2010). Lignin production and lignification of plants is mostly accompanied by an increase in the activity of PODs when plants are responding to environmental stimuli, such as wounding and pathogen attacks (Chittoor et al., 1997; Passardi et al., 2006). To resist various stresses, plants may modify lignin content and/or lignin composition in the plant body (Bonello and Blodgett, 2003; Moura et al., 2010). Lignin induction occurs in the infected plants, in which a mechanical barrier might diminish the probability of pathogen invasion.

Lignin production is mediated by the coordinated expression of several enzymes in the monolignol pathway (Boerjan et al., 2003; Weng and Chapple, 2010). *CCR*. *CAD*, and *POD* genes have been cloned and characterized from plant model species such as *Arabidopsis* and poplar (Lauvergeat et al., 2001; Li et al., 2001; Welinder et al., 2002; Kim et al., 2004) and functional studies have been carried out by evaluating stable transgenic plants (Whetten et al., 1998; Anterola and Lewis, 2002). Down- and up-regulation of *FaCCR*. *FaCAD*, and *FaPOD* gene expression in strawberry fruit have not been reported, yet.

Only recently, examination of the transcriptome, coupled with metabolite profiling analysis of different commercial *F. x ananassa* varieties revealed *FaPOD27* (FaPRX27) as a gene associated with lignin biosynthesis and firmness in strawberry fruit (Ring et al., 2013). Since lignin-related genes have also been detected as differentially expressed in firm fruits in comparison to soft fruits (Salentijn et al., 2003) we investigated lignin formation and composition and its contribution to firmness in strawberry fruit. Lignin biosynthesis genes were isolated from *F. x ananassa* and functionally characterized by biochemical analyses of the encoded proteins as well as by reverse genetics approaches involving agroinfiltration of RNAi and overexpression constructs. Agroinfiltration of strawberries provides an easy and rapid approach to study gene functions (Hoffmann et al., 2006; Schwab et al., 2011).

## Materials and methods

### Plant materials and chemicals

*Fragaria* × *ananassa* cv. Elsanta and transgenic strawberry plants with an antisense *CHS* gene (*F. × ananassa* cv. Calypso; Lunkenbein et al., 2006. *CHS^−^*) were maintained under greenhouse conditions (16/8 h, light/dark period). All chemicals, solvents, and reference compounds were purchased from Sigma-Aldrich (Munich, Germany), Fluka (Munich, Germany), Merck (Darmstadt, Germany), Roth (Karlsruhe, Germany), or Baker (Austin, TX, USA) unless otherwise stated.

### Gene cloning

Total RNA of ripe fruit was extracted as described (Liao et al., 2004). cDNAs as PCR templates were obtained by reverse transcription M-MLV RT H(-) (Promega, Mannheim, Germany) using a random hexamer primer. In order to amplify alleles of the *FaCCR* or *FaCAD* genes, degenerated primers were designed based on deduced amino acid sequences of *CCR* and *CAD* in the GenBank database (www.ncbi.nlm.nih.gov). Since the N- or C-terminal ends of the deduced amino acid sequences of *POD* genes from different species (Welinder et al., 2002; Passardi et al., 2004a) are highly variable, specific primers of *FaPOD* were designed based on the full-length coding sequence of *POD* (*Fragaria vesca*) obtained from the *Fragaria Vesca* Genome Browser database (https://strawberry.plantandfood.co.nz/index.html). The isolation of a full-length coding region sequence of *FaCCR*. *FaCAD*, and *FaPOD* was performed by PCR using a high-fidelity *Pfu* DNA polymerase and its degenerated or specific primers (Table S1). Positive clones were identified by sequencing. *FaPOD27* was isolated as described (Ring et al., 2013).

### Construction of heterologous expression plasmids

After sequencing confirmation, PCR fragments of *FaCCR* and *FaPOD* were amplified by PCR using designed primers (Table S1), based on the full-length coding region, with restriction sites. PCR fragments of the *FaCCR* and *FaPOD* cut with *Bam*HI and *Sma*I were subcloned into a *Bam*HI-*Sma*I cut pGEX-4T-1 vector (GE Healthcare, Munich, Germany), in frame with a coding region for an N-terminal GST (glutathione S-transferase) tag. In addition, the full-length coding region of *FaCAD* was constructed in pET-29a(+) (Novagen, Darmstadt, Germany) as PCR template DNA. The amplified PCR products of *FaCAD* cut with *Bam*HI and *Xba*I were subcloned into a *Bam*HI-*Xba*I cut pYES2 vector (Invitrogen). The resulting plasmid was GST-FaCCR, GST-FaPOD, and pYES2-CAD, respectively. Additionally, the resulting plasmid GST-FaPOD27 was provided by Ring et al. (2013). The full-length coding region sequences of *FaCCR* (accession no. JX290510), *FaCAD* (accession no. JX290511), *FaPOD* (accession no. JX290512), *FaPOD27* (accession no. JX290513) were submitted to the GenBank database.

### Construction of ihpRNA and overexpression plasmids

To prepare vectors for intron-hairpin RNA-mediated gene silencing, PCR products of *FaCCR*. *FaCAD*, or *FaPOD* were amplified by PCR using primers (Table S1) and a high-fidelity Phusion DNA polymerase to introduce *Nhe*I and *Spe*I sites with blunt-end PCR fragments. Then, a 300-bp PCR fragment was digested with *Spe*I and cloned into the compatible site of a *Spe*I-*Ecl*136II pSKAscI35SIntTER vector containing one intron (AY158836, nucleotides 4886–4993) to produce an intermediate pSKAscIA. Afterwards, the same PCR products were additionally digested with *Nhe*I and *Spe*I and cloned into a compatible *Xba*I site of the pSKAscIA vector to produce a pSKAscIAS vector. Finally, the sense- and antisense-mediated fragments were cut *Asc*I from the pSKAscIAS vector and then cloned into the same sites of a binary pBIAscI vector to produce the resulting plasmid pBI-*FaCCRi*, pBI-*FaCADi*, and pBI-*FaPODi*. In addition to construct overexpression vectors, a full-length coding sequence of *FaCCR*. *FaCAD*, or *FaPOD* was amplified by PCR using the Phusion DNA polymerase and primers (Table S1). PCR fragments cut by *Bam*HI and *Sma*I were cloned into the same sites of the binary vector pBI121 to produce the resulting plasmid pBI-*FaCCR*, pBI-*FaCAD*, and pBI-*FaPOD*.

### Heterologous protein expression and protein purification

A bacterial culture *E. coli* BL21 (DE3) pLysS (Novagen, Darmstadt, Germany) containing GST-FaCCR or GST (control) was grown in LB with appropriate antibiotics at 37°C, with shaking at 150 rpm. When OD_600_ reached 0.5–0.6, IPTG was added to a final concentration of 0.2 mM to the bacterial culture at 16°C, with shaking at 150 rpm for 16–18 h. GST-FaCCR was purified using the GST Bind resin (Novagen, Darmstadt, Germany) according to the manufacturer's instructions. In addition, the resulting plasmid GST-POD was transformed into *E. coli* Rosetta (DE3)pLysS cells (Novagen, Darmstadt, Germany). The recombinant protein was expressed and isolated as described (Ring et al., 2013). Besides, pYES2-FaCAD was transformed into yeast cells (*S*. *cerevisae* INVSc.1) using the *S*. *c*. EasyComp Transformation Kit (Invitrogen, Darmstadt, Germany). Positive clones were confirmed by PCR screen, and inoculated in a SC-U selective medium plus 2% galactose to express pYES2-FaCAD in yeast cells. Protein extraction was carried out according to the manufacturer's instructions. The supernatant was used for enzymatic assays. Expression and purification of FaPOD27 was performed as described (Ring et al., 2013).

### Biochemical characterization of GST-FaCCR

Kinetic parameters were determined spectrophotometrically according to Wengenmayer et al. (1976). The substrates (cinnamoyl-, *p*-coumaroyl-, caffeoyl-, and feruloyl-CoA) were enzymatically synthesized as described by Beuerle and Pichersky (2002). To determine pH and temperature optimum, FaCCR activity using feruloyl-CoA as substrate was measured spectrophotometrically and by LC-UV-ESI-MS^n^ analysis, respectively.

### Identification of reaction products by LC-UV-ESI-MS^n^

To verify the identity of reaction products, a FaCCR reaction mixture consisting of 100 mM sodium phosphate (pH 6), 0.1 mM of NADPH, 70 μM of hydroxycinnamoyl-CoA, and 2 μg of purified GST-FaCCR was incubated at 25°C for 20 min. The reaction was stopped by the addition of 25 μl of acetic acid. Following centrifugation at top speed for 10 min, the clear supernatant was analyzed by LC-UV-ESI-MS^n^. In addition, a pYES2-CAD reaction mixture containing 100 mM of sodium phosphate (pH 6.5), 200 μM of NADPH, 100 μM of coniferaldehyde, and 10–100 μg of crude protein was incubated at 30°C for 30 min. The reaction mixture was extracted twice with an equal volume of ethyl acetate by vortexing for 30 s. After centrifugation at top speed for 1 min, ethyl acetate extracts were dried by Speed-Vac (Thermo-Scientific, Dreieich, Germany). For FaPOD and FaPOD27, 300 μl of the reaction mixture, that included 50 mM of sodium tartrate buffer (pH 3.5), 50 μL of substrate (18 mM), and 50 μl of crude proteins (1.9 mg), was incubated at room temperature. 135 μM of H_2_O_2_ (a 30% solution) was added to the mixture at 1-min intervals, one at a time for a total of 4 mM of H_2_O_2_ (Ward et al., 2001). After 30 min, the reaction mixture was extracted as described above. The dried samples were dissolved with 30 μl of 50% methanol (v/v) and used directly for LC-UV-ESI-MS^n^ analysis.

### cDNA synthesis and qRT-PCR analysis

Total RNA was treated with RNase-free DNase I (Fermentas, St. Leon-Rot, Germany) to remove the genomic DNA, according to the manufacturer's instructions. cDNA was synthesized from 1 μg of DNase I-treated total RNA using the M-MLV RT H(-) and 1 μl of 50 μM random hexamer primer according to the manufacturer's instructions. Real-time polymerase chain reactions were performed in a 96-well reaction plate with a StepOnePlus™ real-time PCR system (Applied Biosystems, Darmstadt, Germany) using SYBR Green to monitor dscDNA synthesis. For all qRT-PCR experiments, gene-specific primers (Table S2) were used for amplifications of target genes. An interspacer gene (Table S2) was used as an internal control for normalized expressing values. For data analysis of all samples, relative gene expression was quantified using the 2^−ΔΔ*CT*^ method (Livak and Schmittgen, 2001) to indicate fold changes of each sample related to the selected reference sample.

### Gene expression studies

The differential expression of lignin biosynthetic genes (*FaCCR*. *FaCAD*. *FaPOD*, and *FaPOD27*) in vegetative tissues (leaves, roots, stems, and runners), flowers, and developed fruits at small green, green white, white, turning, and red stages was investigated. All samples were collected from *F*. × *ananassa* cv. Elsanta and immediately frozen in liquid nitrogen as well as stored at −80°C until used.

### Wounding and pathogen treatments

To simulate wounding, a single fruit of *F*. × *ananassa* cv. Elsanta, at the turning stage, was infiltrated throughout the entire fruit with MMA medium (Hoffmann et al., 2006) by using a sterile 1-ml hypodermic syringe. Wounded and untreated control fruits were harvested at different times (12 min, 6, 12, 24, and 48 h). In addition to simulate pathogen infection in a fruit, *F. x ananassa* cv. Elsanta fruit in the turning stage was infiltrated throughout the entire fruit with a suspension of *Agrobacterium* AGL0. Samples were collected at different times (1, 3, 6, 12, 24, 48, and 96 h). All treated fruits remained attached to the plant until harvested. Wild-type (untreated) fruits were used as controls and harvested at the same time. All samples were immediately frozen in liquid nitrogen and stored at −80°C.

### Infiltration of fruits through *Agrobacterium*

All constructs were transferred into *Agrobacterium* AGLO strain (Lazo et al., 1991). Infiltration of strawberry fruit was carried as described (Hoffmann et al., 2006). To assess the effects of independent down-regulation and up-regulation of lignin biosynthetic genes on *F*. × *ananassa* cv. Elsanta and cv. Calypso (CHS^−^) fruits, strawberry fruits of the white developmental stage were injected with *Agrobacterium* suspensions. *Agrobacterium* carrying a pBI-*FaCCRi*, pBI-*FaCADi*, and pBI-*FaPODi* construct were used to down-regulate lignin genes whereas *Agrobacterium* suspensions carrying a pBI-*FaCCR*, pBI-*FaCAD*, and pBI-*FaPOD* construct were applied to up-regulate lignin genes. In addition, fruit of the white developmental stage was injected with a mixture of *Agrobacterium* harboring pBI-*FaCCRi*, pBI-*FaCADi*, and pBI-*FaPODi* constructs to co-down-regulate lignin genes (named pBI-Si3) and with *Agrobacterium* containing pBI-*FaCCR*, pBI-*FaCAD*, and pBI-*FaPOD* constructs to co-up-regulate lignin genes (named pBI-O3). All injected fruits remained attached to the plant until harvested (14 days).

### Fruit firmness and lignin histochemical staining

Fruit firmness was determined by a TA-XT2i texture analyzer (Stable Micro Systems, Godalming, Surrey, UK) according to Bourne (2002), Singh and Reddy (2006). The measuring force was made with a probe of 0.5 mm in diameter to penetrate the surface of the fruit. Each fruit was penetrated at a speed rate of 1–10 mm/s. Based on the bio-yield point, the maximum of force developed during the measurement was recorded and expressed in Newtons (N). Each fruit was measured twice on the two opposite sides of the fruit. The penetrated fruit was frozen in liquid nitrogen immediately, and stored at −80°C until used. In addition to Wiesner staining, lignified tissues were visualized according to the method of Blanco-Portales et al. (2002).

### Enzyme extraction and assays

Enzyme extraction was performed according to Chabannes et al. (2001). Briefly, 200 mg of frozen powder (de-achened fruit) was added to 1 ml of extraction buffer consisting of 0.1 M of Tris-HCl (pH 7.5), 2% PEG 6000 (w/v), 5 mM of DTT, and 2% PVP K30 (w/v). Crude protein was extracted by vortexing for 30 s and incubated on ice for 5 min. After centrifugation at 13,200 rpm for 10 min at 4°C, the supernatant was used for the measurement of enzyme activity. In a total volume of 0.5 ml, a FaCCR reaction consisted of 100 mM of sodium phosphate (pH 6), 100 μM of NADPH, 100 μM of feruloyl-CoA, and 30 μg of crude protein and was incubated at 25°C for 30 min. A FaCAD reaction contained 100 mM of sodium phosphate (pH 6.5), 200 μM of NADPH, 100 μM of coniferaldehyde, and 30 μg of crude protein and was incubated at 30°C for 30 min. Then, the reaction mixture was extracted with ethyl acetate, the solvent was removed and the residue re-dissolved with 30 μl of 50% methanol (v/v) and analyzed by LC-UV-ESI-MS^n^.

### Extraction for metabolite analysis

Two hundred and fifty mg of frozen powder (de-achened fruit) was dissolved in 500 μl of methanol containing 50 mg of biochanin A as an internal standard. The mixture was extracted by vortexing for 1 min, sonicating for 5 min, and centrifuging at 13,200 rpm for 10 min. The supernatant was transferred into a fresh tube and 500 μl of methanol was added to the original tube to extract the residue for a second time. The combined supernatant was dried by Speed-Vac, re-dissolved with 35 μl of water (LC-MS quality), followed by vortexing for 1 min and sonicating for 3 min. After centrifugation at 13,200 rpm for 10 min, the supernatant was used for metabolite analysis by LC-UV-ESI-MS^n^.

### Lignin content

The cell wall from 250 mg of frozen powder (de-achened fruit) was prepared according to the method of Meyer et al. (1998) and Franke et al. (2002). Afterwards, the lignin content of sample was determined by thioglycolic-acid assay. Each sample was mixed with 750 μl of distilled water, 250 μl of 37% HCl, and 100 μl of thioglycolic acid (Sigma-Aldrich, Steinheim, Germany) and incubated at 80°C for 3 h. Subsequent steps were described by Campbell and Ellis (1992). Finally, the insoluble lignin was dissolved in 1 ml of 1 M NaOH. The absorbance of the lignin sample was determined spectrophotometrically at 280 nm. The amount of lignin was calculated from a linear calibration curve (0–20 μg) with lignin hydrolytic (Sigma-Aldrich, Steinheim, Germany).

### Lignin composition

De-achened strawberry fruits (120 ± 20 g) were cut into small pieces and homogenized using an Ultra Turrax (IKA® Works Inc. Wilmington, NC, USA). Insoluble material was washed until the supernatant was determined to be neutral using pH indicator paper. Finally, 10 ml of ethanol (99%) was added to each sample. Following centrifugation at 5100 rpm for 15 min, the pellets were placed at 37°C for drying, overnight. Subsequently, isolation of lignin was performed by the method of Evtuguin et al. (2001), and the resulting lignin was subjected to thioacidolysis (Robinson and Mansfield, 2009). Thioacidolysis products of the samples were analyzed by GC-MS.

### LC-UV-ESI-MS^n^

A Bruker Daltonics esquire 3000^plus^ ion trap mass spectrometer (Bruker Daltonics, Germany) connected to an Agilent 1100 HPLC/UV system with a quaternary pump (Agilent Technologies, Germany) was used. Separation was achieved on a Phenomenex® reversed phase column (Luna 3 μm C18(2) 100 Å 150 × 2 mm). Metabolites, CCR, and POD assays were analyzed using a linear gradient at a flow rate of 0.2 mL min^−1^ for 65 min. The mobile phase A was water with 0.1% formic acid and B was methanol with 0.1% formic acid. The gradient condition was 0–50% B, 0–30 min, then changed to 50–100% B, 30–35 min, 100% B, 35–50 min, 100–0% B, 50–55 min, and equilibrated to 0% B, 55–65 min. The UV detection wavelength was 340 (CCR activity), 320 (POD activity), and 280 nm (metabolite analysis). To determine CAD activity, the program was run a flow rate of 0.1 mL/min with 60% A and 40% B for 30 min at 260 nm. All acquisitions were performed in the positive and negative ionization mode. Capillary voltage was set to −4000 V and the end plate to −500 V. Nitrogen was used as nebulizer gas (30 psi) as well as drying gas at 330°C and 9 L min^−1^. Full-scan mass spectra were acquired from *m/z* 100 to 800 range with a scan resolution of 13,000 m/z/s until the ICC target reached 20,000 or 200 ms, whichever was achieved first. Helium was used as collision gas with 1.0 V collision voltage. Identity of metabolites was confirmed by comparing the retention times, mass spectra, and product ion spectra of the different compounds in the extracts with those of the reference compounds (Hoffmann et al., 2006). In addition to qualitative identification based on fragmentation patterns and retention times each compound was quantified by using QuantAnalysis 1.5. All results were normalized against the internal standard and expressed as mg-equ. kg^−1^ (Hoffmann et al., 2006).

### GC-MS

GC-MS analysis was carried out on a Thermo Finnigan Trace DSQ mass spectrometer coupled with a Thermo Finnigan Trace GC 2000 Ultra with a split injector (1:10). Samples were separated on an Rtx® 5ms fused-silica capillary column (15 m × 0.25 mm, *df* = 0.25 μm) (Restek, Bad Homburg, Germany). The column temperature was held at 90°C for 3 min, then increased to 260°C at a rate of 5°C min^−1^, and finally maintained at 260°C for 15 min. The carrier gas was helium with 1.1 mL min^−1^. The EI-MS ionization voltage was 70 eV (electron impact ionization). The ion source was maintained at 250°C and the interface temperature was at 280°C. GC-MS analysis of trimethylsilylated extracts from treated fruits showed compounds with major ions at *m*/*z* 239, 266, 299, and 57 that eluted at retention times of 27.5, 29, 30.4, and 23.3 min. Fragment ions and retention times correspond to H-, G-, S- monomers that were liberated by thioacidolysis and to the internal standard docosane (I.S., Fig. S1). Lignin breakdown units of each lignin sample were quantified using Xcalibur software (version 1.4). The H-, G-, and S- monomers were calculated based on the peak area of prominent ions related to that of the internal standard docosane for normalization.

### Box plots and statistical analysis

The numerical values from different treatments were transformed into box-whisker plot graphics using the software package R (www.r-project.org). Wilcoxon-Mann-Whitney *U*-test was used for statistical analysis (Hart, 2001; Hoffmann et al., 2006) due to biological variation of each group. The statistical value (*P-value*), based on data of two groups, was calculated by the Wilcoxon-Mann-Whitney *U*-test with a non-parametric statistical analysis using the software package R. *P*-value was used to determine whether or not significant differences existed between treatments.

## Results

### Characterization and heterologous expression of FaCCR, FaCAD, FaPOD, and FaPOD27

The open reading frame (ORF) of *FaCCR* is 1017 bp, encoding a protein of 339 amino acids with a calculated molecular mass of 37.3 kDa and an isoelectric point (pI) value of 6.12. Two conserved motifs, NWYCY, which is putatively involved in the catalytic site of CCRs (Pichon et al., 1998) and a second motif, which is the NAD/NADP(H) binding site in the N-terminal portion of CCRs (Lacombe et al., 1997) are present in amino acid sequences of FaCCR (Fig. S2). To determine the biochemical function of FaCCR, the full-length coding region sequence of *FaCCR* was heterologously expressed in *Escherichia coli* (Fig. S3). The purified recombinant GST-FaCCR showed a pH optimum at 6 in sodium phosphate buffer and a temperature optimum at 25°C (Fig. S4). The protein exhibited Michaelis-Menten kinetics with apparent *K_m_* values of 16.11 (feruloyl-CoA), 25.48 (caffeoyl-CoA), and 24.75 μM (*p*-coumaroyl-CoA). The *K*_cat_/*K_m_* ratios revealed that GST-FaCCR had a high preference for feruloyl-CoA (1.45 10^−4^ s^−1^ μM^−1^; Table 1). In addition to LC-UV-ESI-MS^n^ analyses of individual enzymatic assays, a GST-FaCCR reaction containing a mixture of equal molar amounts of three substrates (caffeoyl-, *p*-coumaroyl-, and feruloyl-CoA), yielded three major peaks that were identified as caffeic aldehyde (3,4-dihydroxycinnamaldehyde), *p*-coumaraldehyde, and coniferaldehyde, respectively. The retention time for each of the three metabolites (Fig. S5) was identical to that of the single product formed by the GST-FaCCR reaction of the individual substrate (Fig. S6). At the same time, the activity of GST-FaCCR toward the different CoA esters was calculated from the peak areas and indicated that feruloyl-CoA (100%) was the preferred substrate compared with both caffeoyl-CoA (3%) and *p*-coumaroyl-CoA (3%) (Fig. S5). These results indicate to a preferential biosynthesis of G units in strawberry fruit.

**Table 1:** **Kinetic properties of the recombinant GST-FaCCR protein**.

**Substrate**	***K_m_* (μM)**	***V*_max_ (nkat mg^−1^ protein)**	***K*_cat_ (S^−1^)**	***K*_cat_/*K*_*m*_ (S^−1^μM^−1^)**
Feruloyl-CoA	16.11 ± 1.39	272.45 ± 15.9	2.34 × 10^−3^	1.45 × 10^−4^
Caffeoyl-CoA	25.48 ± 0.82	3.75 ± 0.24	2.93 × 10^−5^	1.15 × 10^−6^
*p*-Coumaroyl-CoA	24.75 ± 1.23	3.12 ± 0.03	2.65 × 10^−5^	1.07 × 10^−6^

*Values are the mean ± SE for three independent assays*.

FaCAD has a 1077-bp open reading frame encoding a protein of 359 amino acid residues with a relative molecular mass of 39 kDa and a pI value of 5.96. FaCAD is highly similar to Fxacad1 (*F*. × *ananassa* cv. Chandler; 98.1% identity) and PtSAD (*Populus tremuloides*, 77.9% identity) which exhibit different substrate preferences (Li et al., 2001; Blanco-Portales et al., 2002). A conserved NAD/NADP(H) binding sites as well as Zn1 and Zn2 binding motifs are present in the three proteins (Fig. S7). Thus, the FaCAD protein appears to be a zinc-dependent alcohol dehydrogenase. In addition, the full-length coding region of *FaCAD* was subcloned into a pYES2 vector and was expressed in yeast cells (*Saccharomyces cerevisae* INVSc.1). LC-UV-ESI-MS^n^ analysis indicated that extracellular FaCAD activity slightly increased when *FaCAD* was expressed in yeast cells and showed that coniferyl alcohol was generated (Fig. S8). Thus, FaCAD may be involved in the regulation of lignin biosynthesis and contribute to the production of G units in strawberry fruit.

The open reading frame of FaPOD is 990 nucleotides in length and encodes a protein of 330 amino acids with a molecular mass of 37.6 kDa and a pI value of 7.7. FaPOD27 has a 990-bp open reading frame corresponding to a 35.1-kDa protein consisting of 330 amino acids with a calculated pI of 8.47 (Ring et al., 2013). The Prosite analysis (http://prosite.expasy.org/) revealed that FaPOD and FaPOD27 (Ring et al., 2013) are plant heme peroxidases. They both contain a putative Ca^2+^ binding domain and belong to the family of class III peroxidases but exhibit a low percentage of identical amino acids (31.9%). The full-length coding region sequences of *FaPOD* or *FaPOD27* were heterologously expressed in *E. coli* and assayed with different substrates. Since ferulic acid and coniferyl alcohol were almost completely oxidized in the presence of H_2_O_2_ and FaPOD27 the products were subjected to LC-UV-ESI-MS^n^ analysis (Fig. S9, Ward et al., 2001; Ring et al., 2013). A dehydrodimer of ferulic acid (M.W. 386 g/mol) and products formed by decarboxylation of a dehydrodimer precursor (M.W. 342 g/mol) were found as major products (Fig. S9). Unlike FaPOD27, these compounds were not produced by FaPOD or control GST. The results indicate that FaPOD27 is presumably associated with polymerization of coniferyl alcohol and to a lower extent of ferulic acid in lignin. Enzymatic activity of FaPOD could not be detected.

### Expression of lignin biosynthetic genes in different parts of the plant

**Figure 2:**
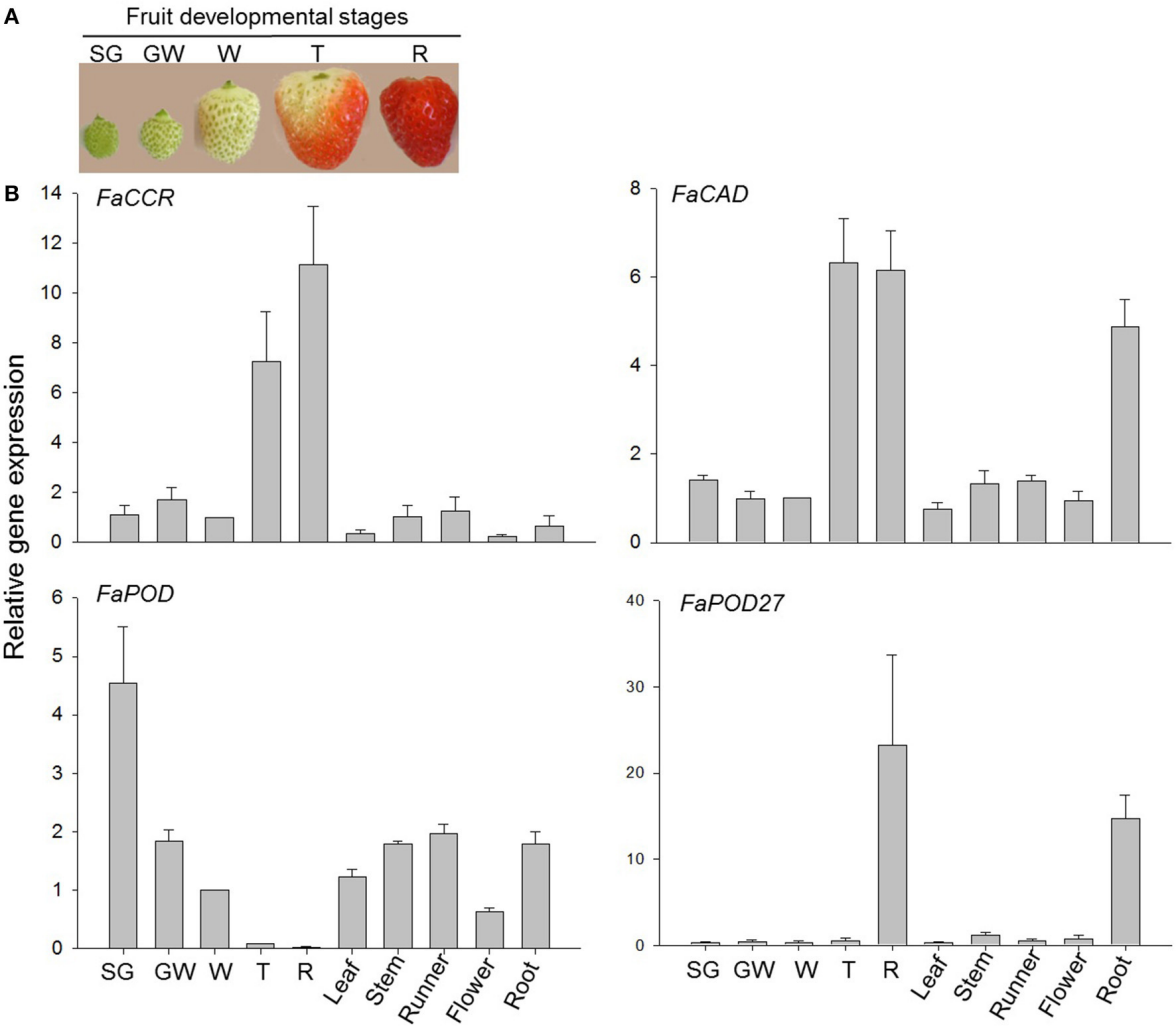
**Relative expression profiles of monolignol biosynthesis genes in vegetative tissues, flowers, and fruit developmental stages of *F*. × *ananassa* cv. Elsanta**. Total RNA was isolated from **(A)** fruit developmental stages at small green (SG), green white (GW), white (W), turning (T), and red (R) after pollination. **(B)** Expression levels of vegetative tissues (leaf; stem; runner; root), flower (F) and fruit developmental stages (SG, GW, W, T, and R) were monitored by qRT-PCR. *FaCCR*. *FaCAD*. *FaPOD* and *FaPOD27* (Ring et al., 2013) were target genes. The white fruit was used as the reference with one for each graph. Values are mean ± *SE* of 5–6 replicates from two sets of cDNAs and are shown as relative changes.

Gene expression studies in *F. x ananassa* cv. Elsanta by qRT-PCR revealed low transcript levels of both *FaCCR*, and *FaCAD* in unripe fruit from SG (small green) to W (white) stage and for *FaPOD27* from SG to T (turning) stage (Figure 2). However, high expression levels of the genes were detected in the mature fruit (T/R for *FaCCR* and *FaCAD* and R in the case of *FaPOD27*). Besides, low mRNA levels of the genes were detected in vegetative tissues and flowers, except for *FaCAD* and *FaPOD27* which were highly expressed in the root. Contrastingly, the highest gene expression level of *FaPOD* was detected in the SG stage and then decreased to almost undetectable levels at the red stage (R). Varied expression levels were detected in vegetative tissues and flowers. Thus, *FaCCR, FaCAD*, and *FaPOD27* genes are strongly expressed in the ripe red fruit, except for *FaPOD*, which is primarily transcribed in the SG fruit.

### Expression of *FaPOD27* is induced in response to *Agrobacterium*

**Figure 3:**
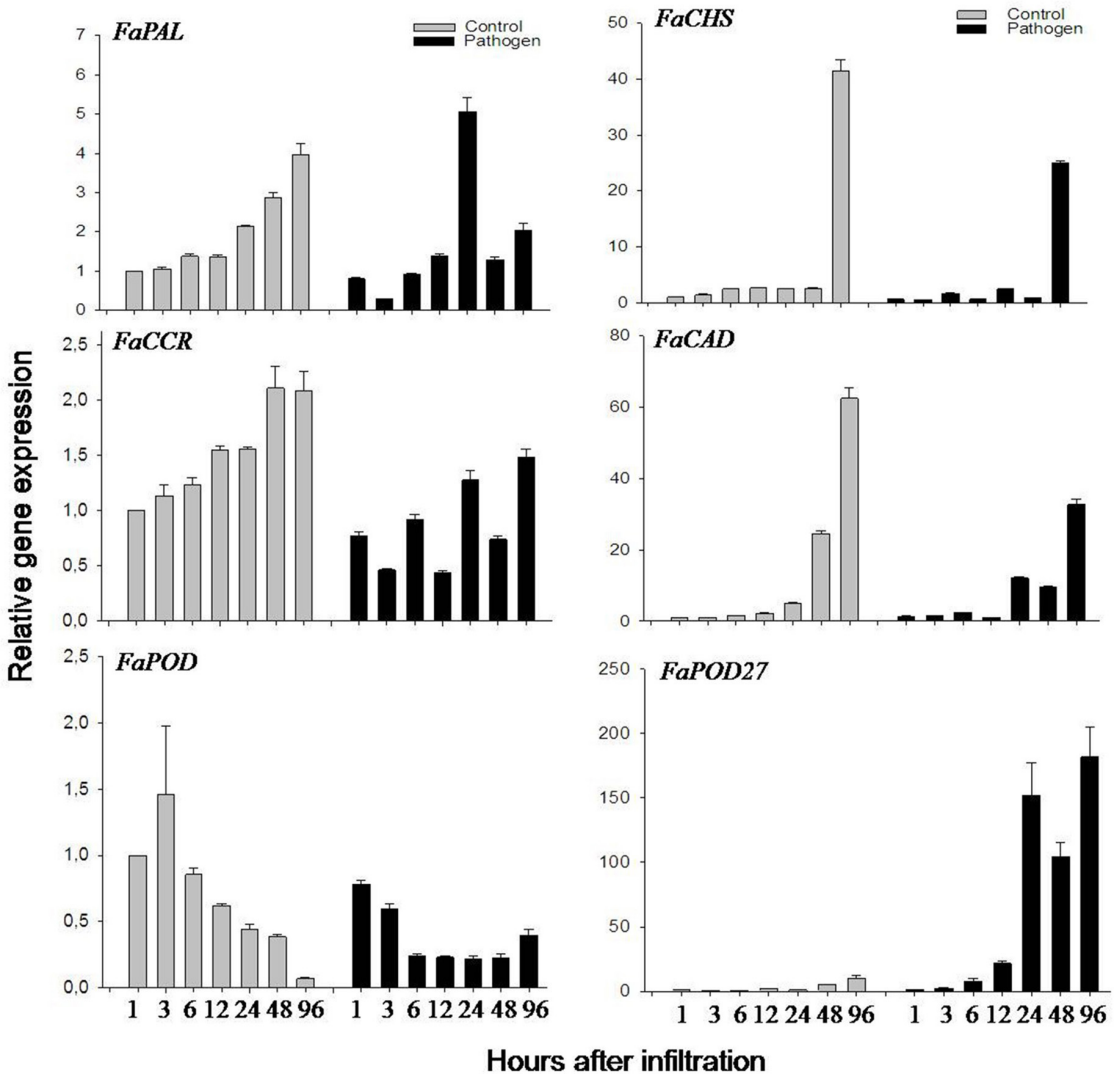
**Relative expression profiles of phenylpropanoid biosynthesis genes of *F*. × *ananassa* cv. Elsanta in response to *Agrobacterium***. Expression levels in control (gray column) and agroinfiltrated fruits (black column) were monitored by qRT-PCR at different time points (1, 3, 6, 12, 24, 48, and 96 h). *FaPAL*. *FaCHS, FaCCR*. *FaCAD*. *FaPOD*, and *FaPOD27* were target genes. The control fruit (1 h) was used as the reference with one for each graph. Values are mean ± *SE* of 2–3 replicates from one fruit and are shown as relative changes.

Lignin biosynthesis is a multi-step pathway, where several genes and enzymes can be induced by various abiotic (plant injuries) and biotic (bacterial infection) stresses (Moura et al., 2010). As agroinfiltration was used in this study to overexpress and down-regulate lignin biosynthesis genes in strawberry fruit, the effect of agroinfiltration on phenylpropanoid metabolism was investigated. Agroinfiltrated fruits were exposed to wounding (perforation of the fruit epidermis by a syringe) and coincidentally to pathogen infection (*Agrobacterium tumefaciens*). Thus, the effect of wounding (injection of MMA buffer) and *Agrobacterium* infection on the expression of putative phenylpropanoid biosynthesis genes was analyzed independently in the fruit. As a result, no significant induction of *FaPAL*. *FaCCR*. *FaCAD*. *FaPOD*, and *FaPOD*27 genes were detected in the wounded fruits, as compared to control (untreated) fruits during 48 h (Fig. S10). Moreover, no significant induction of *FaCHS, FaCCR*. *FaCAD*, and *FaPOD* genes were detected in the fruits exposed to *Agrobacterium*, as compared to control fruits during 4 days (Figure 3). It even appeared that their expression levels were reduced upon agroinfiltration. However, *FaPAL* transcripts were transiently induced at 24 h. In contrast, *FaPOD*27 transcript levels in the fruits injected with *Agrobacterium* gradually started to increase at 6 h and reached a high level at 96 h which was more than 180-fold higher than in control fruit (Figure 3). Also, *FaPOD*27 transcripts were constitutively induced more than 1500-fold within 1–10 days in fruits exposed to *Agrobacterium* (Ring et al., 2013). Thus, *FaPOD*27 transcripts are strongly up-regulated in fruits as a response to infection with *Agrobacterium*, suggesting that this gene plays an important role in pathogen defense.

### Effect of wounding and pathogen infection on fruit firmness

**Figure 4:**
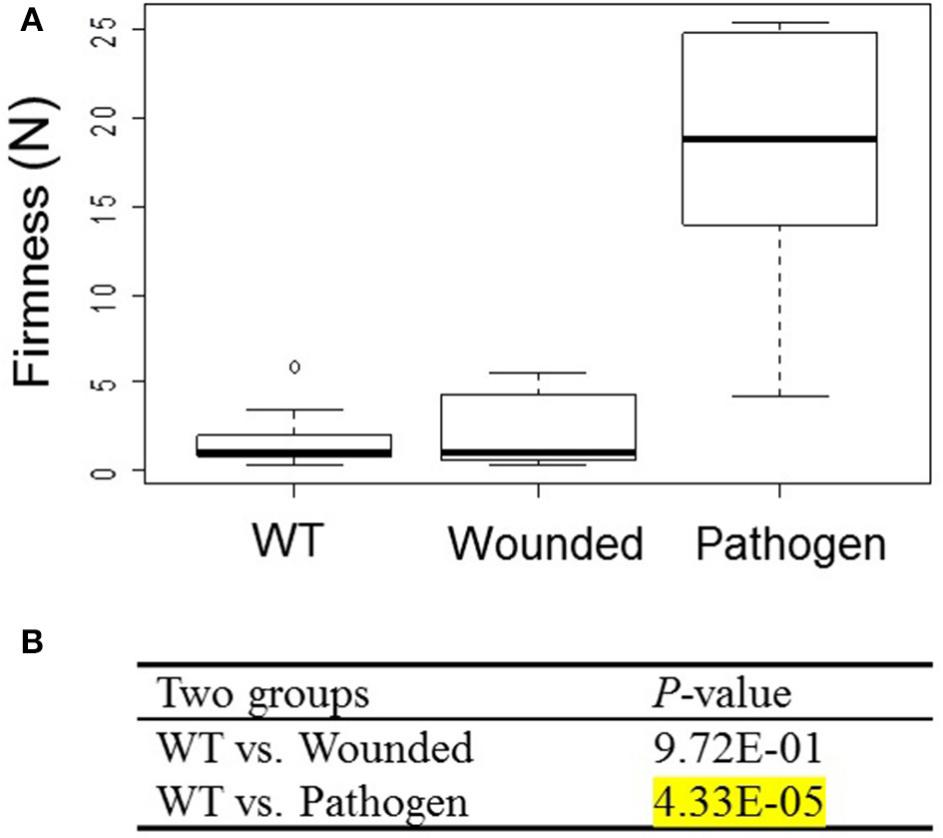
**Effect of wounding and pathogen infection on fruit firmness of *F*. × *ananassa* cv. Elsanta**. **(A)** WT (Wild type; *n* = 10); fruits infiltrated with MMA medium (wounded; *n* = 12) and *Agrobacterium* (pathogen; *n* = 10). **(B)** The Wilcoxon-Mann-Whitney *U*-test was used for a non-parametric comparison of two groups from WT and wounded or pathogen-infected fruits. Values showing statistically significant increased levels (*P* < 1.00E-02) are marked by a yellow background.

To test whether strawberry fruit respond to stress with increased firmness, fruits were either infiltrated with MMA medium (wounding), or infiltrated with *Agrobacterium* (pathogen-infection). Fruit firmness was measured at 14 days after infiltration (Figure 4). Fruit firmness was significantly increased (*P* = 4.33E-05) in fruits infiltrated with *Agrobacterium*, but not in wounded fruits (*P* = 9.72E-01) as compared to untreated WT fruits. Both wounded and wild-type fruits were soft, whereas fruits infiltrated with *Agrobacterium* tended to be hard. The result suggests that fruit firmness significantly increased following infiltration with *Agrobacterium*.

### Downregulation and overexpression of lignin biosynthesis genes by ihpRNA and overexpression constructs in fruit

**Figure 5:**
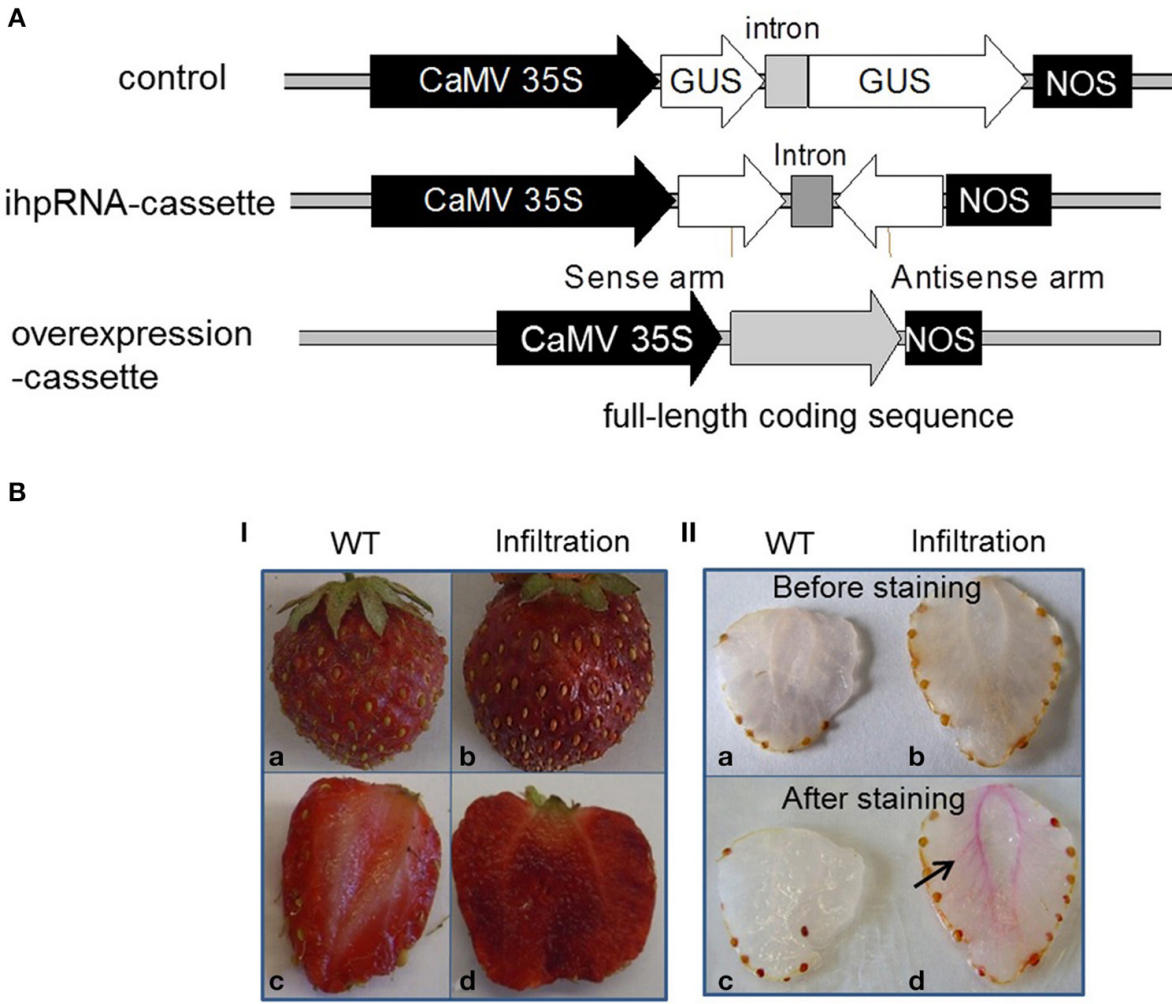
**Schematic diagram of constructs (A), and phenotype (I) and lignin staining (II) of wild-type and infiltrated fruits (B). (A)** All constructs are in the binary vector pBI121. CaMV 35S, the 35S promoter of *cauliflower mosaic virus*; intron, the second intron of strawberry *FaQR* gene (Raab et al., 2006); NOS, terminator of the nopaline synthase gene. A control construct (pBI-intron) contained a *GUS* gene interrupted by an intron; an ihpRNA-cassette construct contained an intron flanked by partial coding sequences (300 bp) of target genes (*FaCCR*. *FaCAD*, or *FaPOD*) in sense and antisense orientations; an overexpression-cassette comprised the full-length coding sequence of target genes (*FaCCR*. *FaCAD*, or *FaPOD*). All constructs were used for agroinfiltration of fruits. **(B)** All photographs were taken 14 days after infiltration. WT (Wild type; a, c) was used as a non-infiltrated fruit. Infiltrated fruits are those injected with *Agrobacterium* suspensions harboring different constructs, as described in **(A)**. **(I)** Phenotypes of WT (a, c) and infiltrated fruits (b, d). **(II)** Cross-sections of WT (a, c; *n* = 2) and infiltrated fruits (b, d; *n* = 2) before and after Wiesner staining. The arrow indicates native lignins in the fruit infiltrated with *Agrobacterium* suspensions harboring different constructs, as described in **(A)**.

In order to manipulate the quantity and quality of lignin in strawberry fruit, we utilized an ihpRNA- and overexpression-cassette with individual *FaCCR, FaCAD*, and *FaPOD* to silence and overexpress *FaCCR, FaCAD*, and *FaPOD* in the fruit. Fruits harboring pBI-intron served as control fruits (Figure 5A). Phenotypic analysis showed that the texture of the fruits injected with different constructs was more solid than that of the untreated fruits (WT). Untreated and treated fruits (pBI-intron control fruits, *FaCCR-*. *FaCAD-*. *FaPOD-*downregulated, *FaCCR-*. *FaCAD-*, or *FaPOD-*upregulated fruits) had retained their red color and were similar in appearance (Figure 5BI). In addition, all treated fruits showed the formation of pink-red colors around vascular bundles within 30 min after lignin staining. In contrast, slight pink-red colors were observed in the untreated fruits (Figure 5BII). This result suggests native lignin around vascular bundles is induced by *Agrobacterium* infiltration. The accumulation of lignin in fruits infiltrated with different constructs (Figure 5A) might be a defense reaction of the fruit to resist *Agrobacterium* invasion.

**Figure 6:**
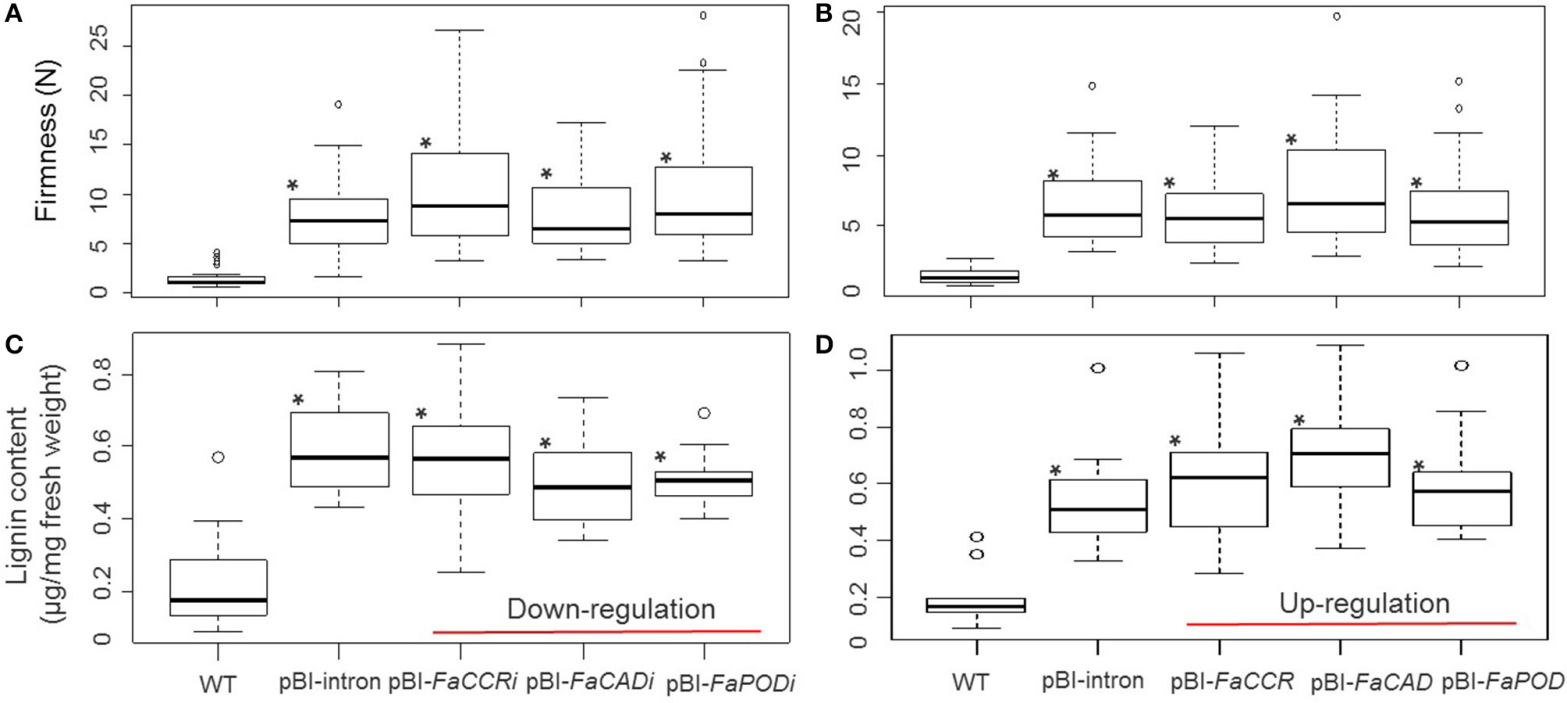
**Fruit firmness (A,B) and lignin content (C,D) of individual *FaCCR-*. *FaCAD-*. *FaPOD*-downregulation (A,C) and -upregulation (B,D) in *F*. × *ananassa* cv. Elsanta. (A)** Firmness of WT (wild type; *n* = 26), control fruits (pBI-intron; *n* = 41), and down-regulated fruits (pBI-*FaCCRi*; *n* = 32, pBI-*FaCADi*; *n* = 30, pBI-*FaPODi*; *n* = 35); **(B)** Firmness of WT (*n* = 30), pBI-intron (*n* = 41), and up-regulated fruits (pBI*-FaCCR*; *n* = 39, pBI-*FaCAD*; *n* = 35, pBI-*FaPOD*; *n* = 43); **(C)** Lignin content of WT (*n* = 14), pBI-intron (*n* = 19), pBI-*FaCCRi* (*n* = 19), pBI-*FaCADi* (*n* = 19), and pBI-*FaPODi* (*n* = 19); **(D)** Lignin content of each group (*n* = 10). The statistical analysis methodology used was the Wilcoxon-Mann-Whitney *U*-test for a non-parametric comparison of two groups. One asterisk (^*^) marked in the box indicates statistically significant increased levels (*P* < 0.01) in comparison with WT and another group.

Both, firmness and lignin content was significantly increased in treated fruits (pBI-intron, pBI-*FaCCRi*, pBI-*FaCADi*, pBI-*FaPODi*, pBI-*FaCCR*, pBI-*FaCAD*, and pBI-*FaPOD* fruits) in comparison with untreated fruits (WT; Figure 6). In addition, comparison of pBI-intron control fruits (agroinfiltrated with a control vector) with the other treated fruits showed that firmness and lignin content were not affected significantly. Thus, when individual *FaCCR*. *FaCAD*, and *FaPOD* were down-regulated and up-regulated in the fruits by agroinfiltration, up-regulation of defense genes such as *FaPOD27* affected fruit firmness and lignin to a much larger extent than the transgenes.

The effect of untreated and treated fruits on the metabolite levels was analyzed by LC-UV-ESI-MS^n^. Major compounds (phenolic acid derivatives, flavonols, anthocyanins, and proanthocyanidins) were quantified. The levels of flavonoids (flavonols, anthocyanins, and proanthocyanidins) were not significantly changed in treated fruits compared with either WT (untreated) fruits or pBI-intron control fruits. In general, the metabolites, whose levels were significantly different between untreated and agroinfiltrated fruits were phenolic acid derivatives (*p*-coumaroyl glucoside/glucose, caffeoyl glucose, and feruloyl glucose, Figure 1 and Fig. S11).

**Figure 7:**
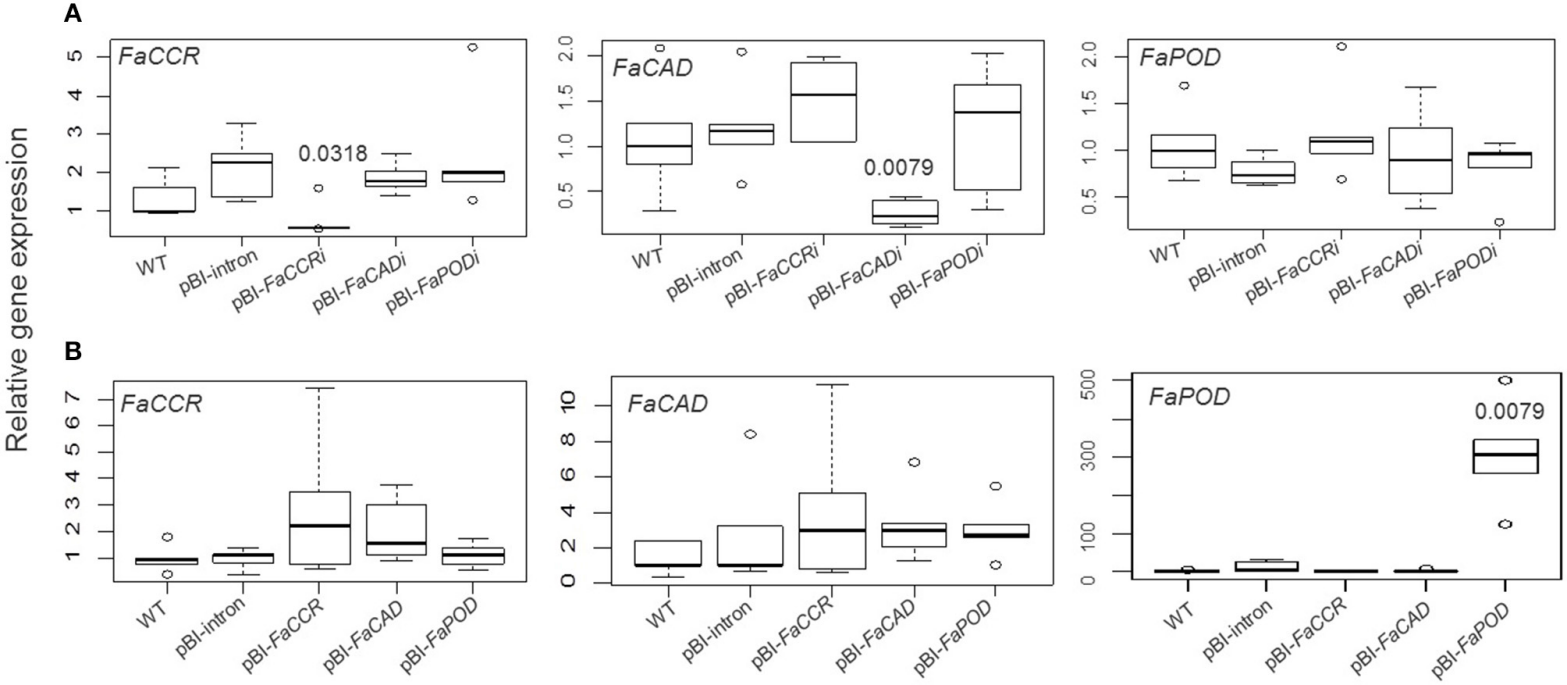
**Relative expression profiles of down- (A) or up-regulated (B) monolignol genes in *F*. × *ananassa* cv. Elsanta**. Total RNA was isolated from a single untreated fruit (wild type; WT), control fruits (pBI-intron), down-regulated fruits (pBI-*FaCCRi*, pBI-*FaCADi*, and pBI-*FaPODi*) **(A)**, and up-regulated fruits (pBI-*FaCCR*, pBI-*FaCAD*, and pBI-*FaPOD*) **(B)**. Expression levels of all samples were monitored by qRT-PCR with specific primers for target genes (*FaCCR*. *FaCAD*, and *FaPOD*) and an interspacer gene. The latter was used as an internal control for normalization. For each box-plot graph, one of the WT group was used as the reference (set to one) and each group contained five biological replicates. The Wilcoxon-Mann-Whitney *U*-test was used for a non-parametric comparison of two groups from pBI-intron and fruits infiltrated with different constructs. Values indicate statistically decreased and increased levels (*P* < 0.05) and are shown in the box.

**Figure 8:**
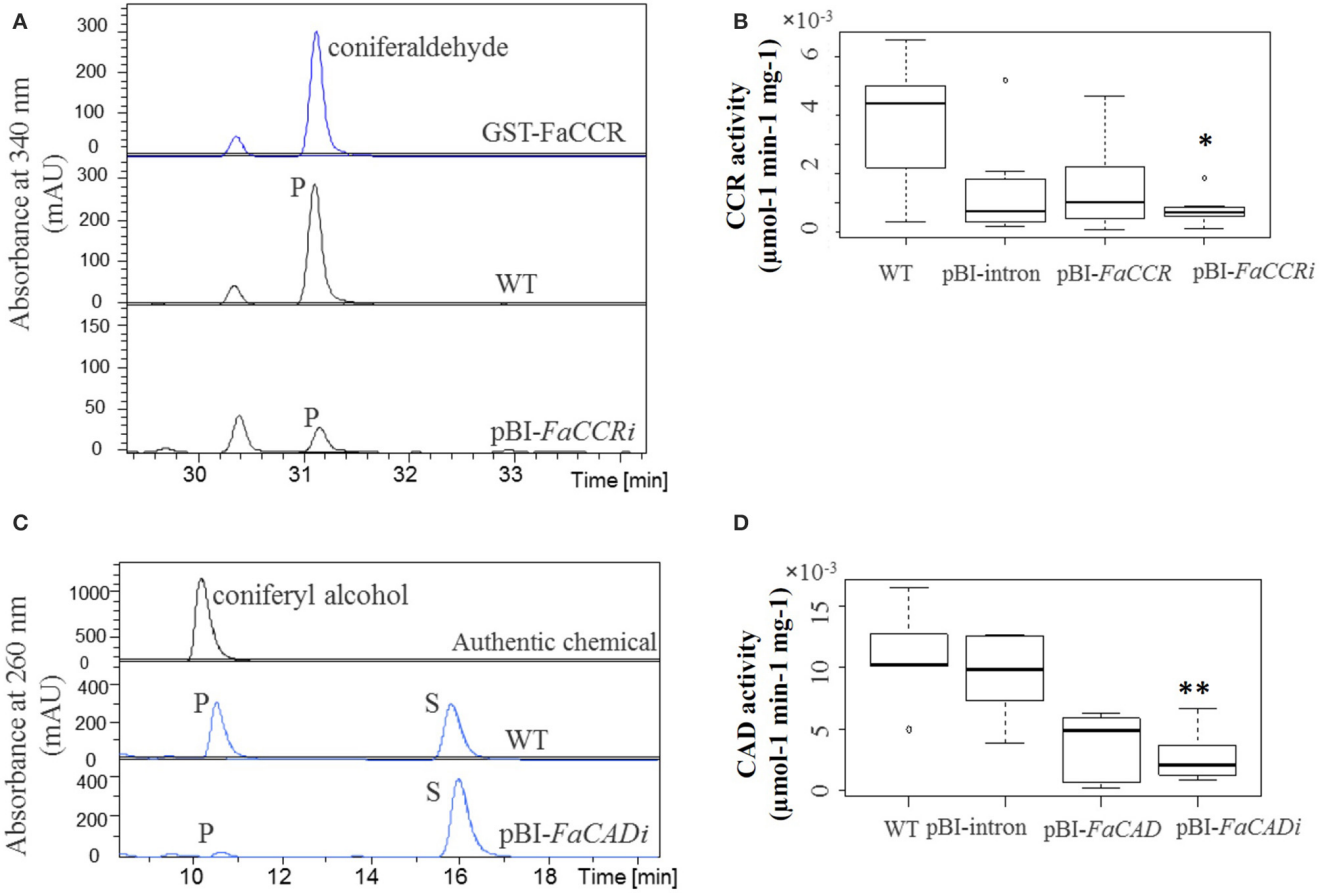
**CCR (A,B) and CAD (C,D) specific activity in wild-type and agroinfiltrated fruits. (A)** Coniferaldehyde (P; products) formed in the reactions was quantified at 340 nm. **(B)** Untreated fruits (WT), control fruits (pBI-intron), pBI-*FaCCR*, and pBI-*FaCCRi*; each group *n* = 7. **(C)** Coniferyl alcohol formed in the reactions (P = coniferyl alcohol; S = coniferaldehyde) was quantified at 260 nm. **(D)** WT, pBI-intron, pBI-*FaCAD*, and pBI-*FaCAD*i fruits; each group *n* = 6. The Wilcoxon-Mann-Whitney *U*-test was used for a non-parametric comparison of two groups. One asterisk (^*^) or two asterisks (^**^) in the box mark statistically significant decreased levels (*P* < 0.02) in comparison with WT and another group (indicated by ^*^), or in comparison with either WT or pBI-intron and another group (indicated by ^**^).

Quantitative real time-PCR was performed to estimate the expression levels of phenylpropanoid biosynthesis genes in *FaCCR-*, *FaCAD-*, *FaPOD*-down-regulated, *FaCCR-*, *FaCAD-*, and *FaPOD*-up-regulated fruits (Figure 7). Transcript levels of both *FaPAL* and *FaCHS* were not significantly changed in these fruits (data not shown). However, in comparison to pBI-intron control fruits, *FaCCR* and *FaCAD* transcripts were significantly decreased (*P* < 0.05) in pBI-*FaCCRi* and pBI-*FaCADi* fruits, respectively (Figure 7A). They were not significantly different in other treated fruits. Thus, the ihpRNA-*FaCCR* and -*FaCAD* construct showed sequence-specific interference with homologous *FaCCR* and *FaCAD* expression in the fruits. However, *FaPOD* transcripts were not significantly affected in pBI-*FaPODi* fruits (Figure 7A) probably due to the already low expression level of *FaPOD* in the ripe red fruit (Figure 2B). In the case of up-regulation of monolignol genes in the fruits, expression levels of *FaCCR* and *FaCAD* were not significantly affected in pBI-*FaCCR* and pBI-*FaCAD* fruits (Figure 7B). *FaCCR* and *FaCAD* transcripts are already abundant in the ripe red stage of WT fruit (Figure 2B) but their levels did not increase in pBI-*FaCCR* and pBI-*FaCAD* fruits (Figure 7B). Interestingly, levels of *FaPOD* transcripts which are rarely expressed in the ripe red stage (Figure 2B) increased significantly (*P* < 0.05) when *FaPOD* was overexpressed in pBI-*FaPOD* fruits (Figure 7B). Besides, the effect of untreated and treated fruits (*FaCCR-* and *FaCAD*-downregulated and -upregulated fruit) on enzyme activities was analyzed by LC-UV-ESI-MS^n^. FaCCR and FaCAD activity was reduced in pBI-*FaCCRi* and pBI-*FaCADi* but also in pBI-FaCCR and pBI-FaCAD fruits (Figure 8) in comparison with untreated WT fruit. The dsRNA produced by pBI-*FaCCRi* and pBI-*FaCADi* can trigger PTGS (post-transcriptional gene silencing) to interfere with homologous *FaCCR* and *FaCAD* expression. Co-suppression of homologous genes can, at least in parts, explain the observation of reduced CCR and CAD activity in pBI-FaCCR and pBI-FaCAD fruits.

To determine lignin composition and estimate proportions of lignin monomers, lignin was extracted from pooled fruits of each treatment and was subjected to thioacidolysis. H-, G-, and S-monomers were not detected in WT (untreated) fruits due to the low yield of lignin produced in these fruits (Table 2). In contrast, H-, G-, and S-monomers were detected in all treated fruits, because higher levels of lignin accumulated in the fruits exposed to *Agrobacterium*. In comparison to pBI-intron control fruits, low amounts of H-monomers were detected in all treated fruits. *FaCAD*-silenced fruits showed a significant reduction of 58% in H-monomers. Levels of G-monomers were significantly reduced by 35, 33, and 32% in the pBI-*FaCCRi*, pBI-*FaCADi*, and pBI-*FaPOD* fruits, respectively. Levels of S-monomers were significantly reduced by 22 and 13% in pBI-*FaCADi* and pBI-*FaPOD* fruits, respectively. However, amount of S-monomers was significantly elevated by 18% in the pBI-*FaCCR* fruits. Remarkably, levels of both G- and S-monomers were significantly reduced in the pBI-*FaCADi* and pBI-*FaPOD* fruits. In addition, *FaCCR*-silenced fruits showed the highest increase in the S/G ratio (1.36 ± 0.12) of lignin, as compared to all treatments (Table 2).

**Table 2:** **Impact of fruits injected with different constructs on lignin monomer composition**.

**WT**	**Relative concentration (%, ML)**			
	**Proportion of thioacidolysis monomers**			
	**H**	**G**	**S**	**S/G ratio**
	**ND**	**ND**	**ND**	**ND**
pBI-intron	0.19 ± 0.04	3.83 ± 0.05	3.65 ± 0.11	0.95 ± 0.04
pBI-*FaCCRi*	0.14 ± 0.09	2.50 ± 0.37^*^	3.38 ± 0.21	1.36 ± 0.12^*^
pBI-*FaCADi*	0.08 ± 0.02^*^	2.55 ± 0.08^*^	2.85 ± 0.17^*^	1.12 ± 0.10
pBI-*FaPODi*	0.13 ± 0.02	3.93 ± 0.01	4.09 ± 0.27	1.04 ± 0.07
pBI-*FaCCR*	0.17 ± 0.03	4.02 ± 0.22	4.29 ± 0.03^*^	1.07 ± 0.05
pBI-*FaCAD*	0.16 ± 0.02	3.41 ± 0.23	4.17 ± 0.44	1.22 ± 0.05^*^
pBI-*FaPOD*	0.10 ± 0.004	2.62 ± 0.01^*^	3.19 ± 0.03^*^	1.22 ± 0.02^*^

For each treatment, fruits (120 ± 20 g) were pooled to extract lignin. Values are the means ± s.e.m. of duplicates of two independent analyses from the pooled lignin. ML, monomeric lignin; ND, not detected. One asterisk (

* *) indicates significant differences by Student t-test (P < 0.01) in comparison with pBI-inron and another treatment*.

### Individual or combined down- or up-regulation of monolignol genes in CHS-deficient (CHS^−^) fruit

**Figure 9:**
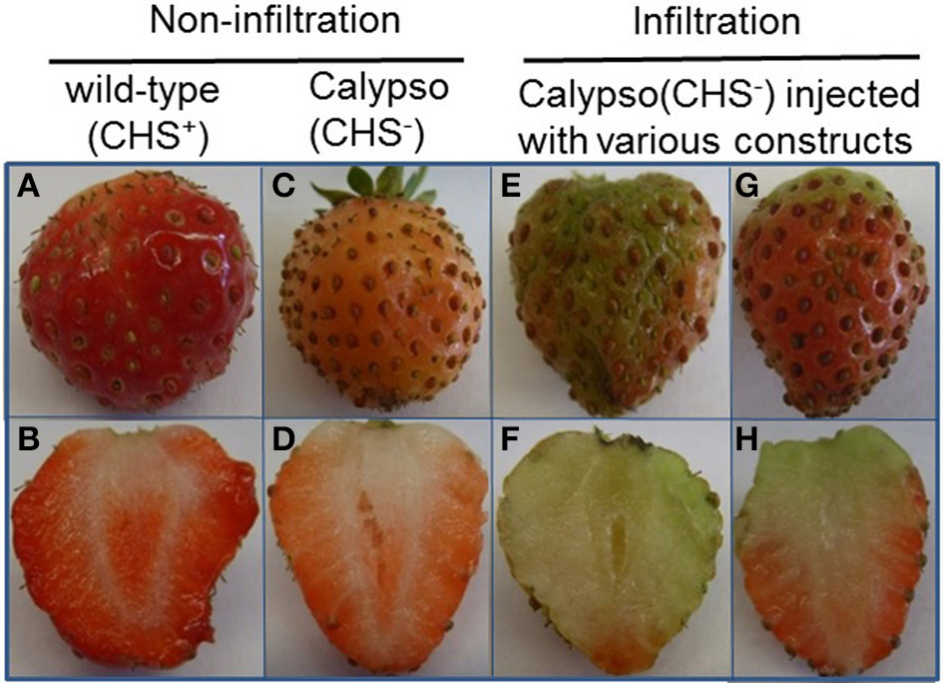
**Comparison of non-infiltrated and infiltrated Calypso (CHS^−^) fruits**. All pictures were taken 14 days after infiltration. Phenotypes of non-infiltrated **(A–D)** and infiltrated fruits **(E–H)** are compared. Non-infiltrated fruits of Calypso with CHS genes (CHS^+^; **A,B**) and Calypso with impaired CHS genes (CHS^−^; **C,D**) are shown. Infiltrated fruits **(E–H)** represent Calypso fruit (CHS^−^) injected with *Agrobacterium* suspensions harboring either pBI-intron, *FaCCR-*. *FaCAD-*. *FaPOD*-ihpRNA, *FaCCR-*. *FaCAD-, FaPOD*-overexpression constructs, or combined pBI-Si3 and pBI-O3 constructs. All agroinfiltrated fruits showed a similar phenotype **(E–H)**.

*p*-Coumaroyl-CoA is the common substrate of CHS, HCT, and CCR enzymes in the phenylpropanoid metabolism (Figure 1). When the *CHS* gene is silenced in fruits, the pool of *p*-coumaroyl-CoA increases (Lunkenbein et al., 2006) and could provide additional precursors (*p*-coumaroyl-CoA) for the synthesis of H-, G-, and S-lignin. In this work, individual and combined down- and up-regulation of monolignol genes was also performed by agroinfiltration in fruits of stable transgenic antisense CHS Calypso (CHS^−^) lines. CHS-deficient fruits injected with different constructs (infiltration) remained white or only slightly red in color, whereas fruits with impaired *CHS* (CHS^−^) turned pink and control wild-type fruits (CHS^+^) developed a red color (Figure 9). Fruits of the CHS^−^ line carry the antisense *CHS* gene that results in lower formation of anthocyanins in comparison to fruits of WT plants but pigment formation is further reduced by agroinfiltration independent of the construct used. In the *CHSi*-deficient background, transcriptional profiles of phenylpropanoid biosynthesis genes in fruits agroinfiltrated to down- and up-regulate genes of the monolignol pathway were quite consistent with the results from Elsanta fruits (Fig. S12 and Figure 7). Levels of *FaCCR* and *FaCAD* transcripts were significantly reduced in *FaCCRi-* and *FaCADi*-silenced *CHS^−^* fruits, whereas *FaPOD* mRNA levels were considerably increased in *FaPOD*-overexpressed fruits (Fig. S12) in comparison to the results in *CHS^−^* controls. An exception was *FaPAL* whose transcript level was remarkably reduced in pBI-FaCADi fruit.

**Figure 10:**
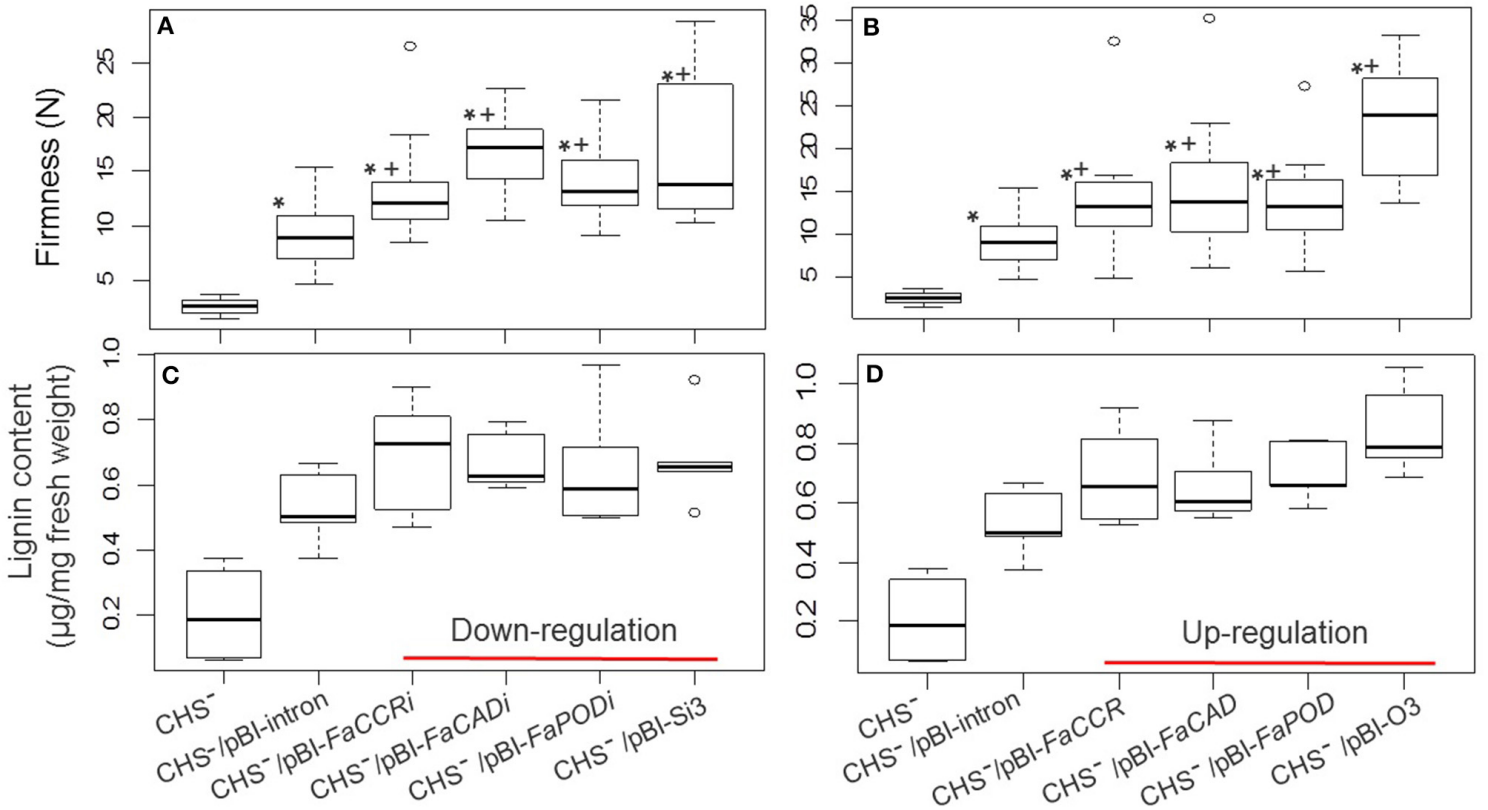
**Fruit firmness (A,B) and lignin content (C,D) of *FaCCR-*. *FaCAD-*. *FaPOD*-downregulation and -upregulation as well as combinations in *F*. × *ananassa* cv. Calypso (CHS^−^)**. **(A)** Firmness of Calypso (CHS^−^; *n* = 15), control fruits (CHS^−^/pBI-intron; *n* = 17), down-regulated fruits (CHS^−^/pBI-*FaCCRi*; *n* = 14, CHS^−^/pBI-*FaCADi*; *n* = 14, CHS^−^/pBI-*FaPODi*; *n* = 17), and combined three genes (CHS^−^/pBI-Si3; *n* = 15). **(B)** Firmness of Calypso (CHS^−^, *n* = 15), control fruits (CHS^−^/pBI-intron; *n* = 17), up-regulated fruits (CHS^−^/pBI-*FaCCR*; *n* = 14, CHS^−^/pBI-*FaCAD*; *n* = 17, CHS^−^/pBI-*FaPOD*; *n* = 15), and combined three genes (CHS^−^/pBI-O3; *n* = 18). **(C,D)** Lignin content of each group (*n* = 5). The Wilcoxon-Mann-Whitney *U*-test was used for non-parametric analysis of two groups. One asterisk (^*^) or a plus (+) in the box marks statistically significant increased levels (*P* < 0.05) in comparison with CHS^−^ and another group (indicated by ^*^), or in comparison with CHS^−^/pBI-intron and another group (indicated by +).

In addition, the effect of down- and up-regulation of monolignol biosynthesis genes on texture and lignin content in CHS^−^ fruits was investigated. The comparison of firmness and lignin content of CHS^−^ (untreated fruit) with CHS^−^/pBI-intron control fruit and the other treated fruits (CHS^−^ fruits agroinfiltrated to down- and up-regulate monolignol genes) showed that fruit firmness and the amount of lignin is primarily affected by agroinfiltration as injection of *Agrobacterium* carrying the control vector already increases firmness and lignin content to high levels (Figure 10).

## Discussion

### Biochemical characterization of FaCCR, FaCAD, FaPOD and FaPOD27

As comparative microarray analyses have provided first evidence for a contribution of lignin in strawberry fruit firmness (Salentijn et al., 2003) we analyzed the role of lignin biosynthesis genes in fruit ripening. The activity of the CCR enzyme has already been observed in several plant species. Our *in vitro* results indicate that FaCCR is optimal at pH 6.0 in a 100 mM sodium phosphate buffer at 25°C. Similarly, soybean CCR activity exhibited an optimum at pH 6.0–6.2 in 100–200 mM citrate buffer at 30°C and was stable at around pH 7.0 (Wengenmayer et al., 1976) whereas eucalyptus CCR activity was optimal at pH 5.3–6.5 in a potassium/sodium phosphate buffer (Goffner et al., 1994). In addition, FaCCR exhibited the highest affinity with feruloyl-CoA. This was quite consistent with other angiosperm CCR proteins, which convert feruloyl-CoA with greater efficiency (Lauvergeat et al., 2001; Li et al., 2005). The poor conversion rate of CCRs with *p*-coumaroyl-CoA explains the low content of H units in the lignin of angiosperms (Li et al., 2005). Feruloyl-CoA and sinapoyl-CoA would be converted into G and S monomers, respectively, *via* an independent or cross route, and eventually, heterologous G-S lignin is formed in angiosperms (Dixon et al., 2001).

FaCAD and Fxacad1 (*F*. × *ananassa* cv. Chandler) proteins share 98.1% amino acid identity. In *in vitro* studies, pYES2-*FaCAD* was successfully expressed in *S*. *cerevisiae* and low amount of coniferyl alcohol product was generated (Fig. S8). Besides, the recombinant Fxacad1 enzyme expressed in *Pichia pastoris* cells exhibited high activity with cinnamyl (100% activity), coniferyl (51.2%), and sinapyl (64.3%) aldehyde (Blanco-Portales et al., 2002). This suggests that FaCAD is a putative NADPH-dependent cinnamyl alcohol dehydrogenase.

Two strawberry peroxidases, FaPOD and FaPOD27, which belong to the group of plant heme peroxidase, have been analyzed in this study. Recombinant FaPOD27 was successfully produced in *E. coli* and crude protein extracts containing FaPOD27 oxidized putative lignin precursors such as coniferyl alcohol, ferulic acid, and caffeic acid, in the presence of H_2_O_2_ into dehydrodimers. The reactivity of FaPOD27 strongly suggests a function of this enzyme in the polymerization of phenolics (Ring et al., 2013). However, recombinant FaPOD was poorly expressed in *E. coli* and remained inactive when tested with putative lignin precursors. Two AGA and AGG codons were found 28 times in the full-length coding sequence of *FaPOD*. Since prokaryotes rarely employ tRNAs for AGA and AGG arginine codons, genes containing these codons can hardly be expressed in regular *E. coli* strains (Hushpulian et al., 2003). However, active *AtPOD33*. *AtPOD34*, and *AtPOD37* proteins have been expressed in eukaryotic host baculovirus-insect cells (Carpin et al., 2001). Peroxidases can be expressed in such hosts, where the native proteins fold correctly and act as functional proteins. This system was not yet used for the heterologous expression of FaPOD. However, *FaPOD* gene expression data suggest a role in early stages of fruit development (Figure 2).

### Fruits in response to *Agrobacterium* attack

Inducing the expression of defense-related genes and coordinating complex interactions between defense-signaling pathways are major factors in activating a defense response against pathogen attack in plants (Rushton and Somssich, 1998). PAL, COMT, 4CL, CAD, and POD showed transiently increased activity in pine cell culture following treatment with an elicitor derived from an ectomycorrhizal fungus (Campbell and Ellis, 1992). The expression of peroxidase genes was induced in both the epidermis and mesophyll tissues in wheat after Bgt attack (Liu et al., 2005). In the present study, expression of *FaPOD27* was clearly induced in fruits after infection with *Agrobacterium*. *FaPOD*27 showed a low basal expression in different tissues but its transcript levels constantly and significantly increased during 10 days upon agroinfiltration (Ring et al., 2013). Importantly, the transcript levels of *FaPOD* remained unchanged after infection by *Agrobacterium*. In plants, a basal level of peroxidase functions probably as housekeeping activity in either elongation or lignification (Passardi et al., 2005). However, induction of POD activity in response to pathogen attack results in increased lignification (Xu et al., 2012) and thus reinforcement of plant cells to prevent the entry of pathogens. Similarly, *FaPOD*27 transcripts might be required to prevent the spreading of *Agrobacterium* in infected fruits. Elevated levels of *FaPOD27* transcripts were detected in all strawberry fruits that were agroinfiltrated (Ring et al., 2013) and subsequently underwent active lignin synthesis (Figure 5BII). However, enzyme abundance and enzymatic activity of FaPOD27 was not determined in agroinfiltrated fruit.

It has also been reported that the expression of various isoenzymes is induced by different stress signals (Lauvergeat et al., 2001). Rice POX8.1 and POX22.3 were largely induced during resistant interactions, but POXgX9 was not induced by either wounding or pathogen (Chittoor et al., 1997). Similarly, *FaPOD* transcript levels remained unchanged although *FaPOD27* transcription was strongly induced upon pathogen infection (Figure 3).

*Agrobacterium*-infected fruits, concomitant with induced *FaPOD27* transcripts levels, clearly showed enhanced firmness and increased lignin content (Figures 4, 6). Likewise, PAL, CAD, and POD activities increased in loquat fruit, concurrently with the increase in lignin content (Cai et al., 2006). Moreover, the abundance of *FaCCR* and *FaCAD* transcripts in the mature fruit (Figure 2B) may supply sufficient monolignols that are oxidized by FaPOD27 to form additional lignin in *Agrobacterium*-infected fruit. These results indicate that increased expression levels of *FaPOD27* provoke the enhanced production of lignin in *Agrobacterium*-infected fruits. Thus, *FaPOD27* is presumably a major factor involved in lignin biosynthesis associated with the defense response.

### Phenotype, fruit firmness, and lignin content

To demonstrate the role of monolignol genes in regulating lignin content in plants, monolignol genes were transiently down-regulated by RNAi and over-expressed in strawberry fruits. In this study, the lignin content of agroinfiltrated fruits expressing down- and up-regulation constructs of monolignol genes equaled the level in the pBI-intron control fruits but was significantly higher than the content in untreated fruits (Figure 6). At the same time, all fruits exhibited normal growth and development (Figure 5BI). When monolignol genes are introduced into plants, transgenic plants show normal or unusual phenotypes, depending on the reduction in lignin content. An abnormal phenotype was observed when total lignin content decreased in CCR-down-regulated tobacco plants (Piquemal et al., 1998) but down-regulation of CAD did not significantly affect total lignin and tobacco plants exhibited a normal phenotype (Ralph et al., 1998). It was assumed that lignification was sufficiently plastic to enable CAD-down-regulated plants to form lignin from hydroxy cinnamylaldehydes (Humphreys and Chapple, 2002).

In addition, in our study, enhanced fruit firmness in agroinfiltrated fruits was always associated with increased lignin production, when compared to wild-type (untreated) fruits (Figure 6). This demonstrates a clear correlation between fruit firmness and lignin content. It has already been shown that lignification and lignin formation is induced by pathogen infection (Reimers and Leach, 1991; Passardi et al., 2005, 2006; Bhuiyan et al., 2009) which is a common behavior in a plant's defense against penetration by pathogens. In this work, lignin staining revealed lignin accumulation mainly in the vascular tissue in treated fruits exposed to *Agrobacterium* (Figure 5BII). It is assumed that deposition of lignin in agroinfiltrated fruits led to reinforced cell walls, resulting in enhanced firmness in the treated fruits.

### Effect of treated fruits on biosynthesis of phenylpropanoid intermediates

Hydroxycinnamic acids accumulate exclusively as glucose ester in strawberry fruit but levels of the glucose ester may reflect the amounts of their corresponding phenylpropanoic acid precursors (Figure 1; Määttä-Riihinen et al., 2004). Increased levels of ferulic acid in wheat plants, exposed to bacterial infection, suggest the induction of a defense response to prevent bacterial infection in these plants (Parrott et al., 2002). Likewise reduced levels of *p*-coumaroyl glucoside/glucose and increased amounts of feruloyl and caffeoyl glucose ester were observed in agroinfiltrated fruits in comparison with untreated controls (Fig. S11), suggesting that coumaric acid is converted to fuel the lignin pathways.

In particular, the level of coumaric acid decreased in *CCR*-down-regulated lines of perennial ryegrass, whereas levels of caffeoylquinic acid, caffeoyl shikimic acid, ferulic acid, and sinapic acid increased in these transgenic plants (Tu et al., 2010). In addition, low levels of *CCR* diverted *p*-coumaroyl-CoA into the synthesis of flavonoids, resulting in the accumulation of flavonol conjugates in CCR-deficient plants (van der Rest et al., 2006; Tu et al., 2010). Consistent with these result, *FaCCR*-silenced fruits contained the least amount of *p*-coumaroyl glucose esters (Fig. S11A). However, the levels of flavonol conjugates were not affected probably because most of the precursors of flavonols have been formed prior to agroinfiltration.

### Restoration of lignin content and modification of monolignol composition

Lignin content and composition play an important role in evaluating the results of genetic biotechnologies. Recent studies have shown that a compensatory mechanism occurs in *CCR-* and *CAD*-down-regulated angiosperms, in which elevated ferulic acid and hydroxyl cinnamaldehyde were incorporated into the lignin polymer (Lapierre et al., 1999; Chabannes et al., 2001; Ralph et al., 2008; Tu et al., 2010; Vanholme et al., 2010). It has been reported that both *p*-coumaric and ferulic acid were cross-linked to cell wall lignin of grasses (Grabber et al., 1991). In the present study, metabolite profiling detected increased levels of caffeic and ferulic acid glucose ester in strawberries injected with *Agrobacterium* (Fig. S11). Therefore, we assumed that elevated levels of ferulic and caffeic acid may be incorporated into lignin to restore its content in treated fruits. In recent years, thioacidolysis marker compounds for the incorporation of ferulic acid and hydroxy cinnamaldehyde into the lignin polymer have been established (Palmer et al., 2008; Ralph et al., 2008) and β-O-4 coupled hydroxyl cinnamalaldehyde structures in lignin of CAD-down-regulated tobacco have been revealed by NMR methods (Chabannes et al., 2001; Ralph et al., 2001). However, ferulic acid was not detected in significant amounts in treated strawberry fruits (Fig. S13) and the aldehyde was not seen in pBI-*FaCADi* lignin after thioacidolysis by GC-MS. Thus, the time period between agroinfiltration and harvest of the fruits is probably too short to incorporate greater amounts of ferulic acid or hydroxy cinnamaldehyde. Similarly, ferulic acid was also not detected in the *Zmccr1^−^* mutant (Tamasloukht et al., 2011). Remarkably, a new thioacidolysis product was detected in *FaCCR*-silenced fruits but remained unknown (Fig. S13).

In addition to reduced activities of target enzymes, *CCR-* and *CAD*-down-regulation in tobacco also affected the proportion of monolignols in the lignin polymer (Chabannes et al., 2001). In CCR-deficient plants such as tobacco, *Arabidopsis*, and maize the flux from feruloyl-CoA to G and S units is significantly decreased (Chabannes et al., 2001; Ralph et al., 2008; Tamasloukht et al., 2011). Similarly, we observed a significant reduction in the relative concentration of G units in *FaCCR*-silenced fruits, as well as G and S units in pBI-*FaCADi* fruits compared with pBI-intron control fruits (Table 2). In an *in vitro* study, GST-CCR was highly specific to feruloyl-CoA (Table 1) which nicely explains the decreased flux from feruloyl-CoA to G units in *FaCCRi* fruits where the level of *CCR* transcripts were significantly reduced (Figure 7A). Besides, the increase in the S/G ratio of lignin in *FaCCR*-silenced fruits was consistent with results obtained from CCR-deficient plants (Chabannes et al., 2001; Tamasloukht et al., 2011). It was showed that the recombinant FxaCAD1 (*F*. × *ananassa* cv. Chandler) enzyme is highly specific for coniferyl and sinapyl aldehydes *in vitro* (Blanco-Portales et al., 2002). This result is consistent with the observation of a decreased flux from coniferyl and sinapyl aldehyde to G and S units, respectively in *FaCAD*-silenced fruits. In addition, enhanced *FaPOD* transcripts may not have a direct effect on total lignin content (Figures 6D, 7B), but levels of G and S units decreased significantly in pBI-*FaPOD* fruits (Table 2). Hence, FaPOD seemed to be functional in a lignin related pathway in *FaPOD*-overexpressed fruit and is proposed as regulator of the lignin composition. Possible explanations are that FaPOD catalyzes a reaction that limits monolignol supply for lignin formation or inhibits the activity of other POD isoenzymes whereby regulating the proportion of G and S units in lignin. The results show that FaPOD activity is related with lignin composition probably in immature fruit where the gene is highly expressed (Figure 2B).

Altogether, variations in the S to G unit ratios of lignin may be attributed to the fact that different monolignol genes were introduced or silenced in the fruits (Table 2) whereas the overall lignin content in all treated fruits may be ascribed to elevated transcript levels of *FaPOD27* (Figure 3). Elevated levels of *FaPOD27* in agroinfiltrated fruits seem to have a much stronger effect on the total lignin content than down-regulation of lignin biosynthesis genes (Figure 7).

### Increased firmness and lignin level in CHS^−^ fruits due to manipulation of monolignol genes

Suppression of the *CHS* gene in strawberry fruit results in an increase in the pool of *p*-coumaroyl-CoA derived metabolites (Lunkenbein et al., 2006) which may serve as precursors for the synthesis of lignin through HCT and CCR activity (Figure 1). Similar to fruits of wild type *F. x ananassa* cv Elsanta, in CHS^−^ fruits of *F. x ananassa* cv. Calypso the increase in firmness upon agroinfiltration of different constructs was also associated with an increase in lignin content (Figure 10). A comparison revealed that fruits agroinfiltrated with silencing and overexpression constructs were firmer than CHS^−^/pBI-intron control fruits (Figure 10).

It has been shown that flavonoid accumulation in *HCT^−^ A. thaliana*, repressed in lignin synthesis, affects auxin transport and plant growth (Besseau et al., 2007). Remarkably, auxin transport can be restored in HCT^−^/CHS^−^ plants due to lower levels of flavonoids in the plants. Restoration of auxin transport also led to increased yields of lignin. In accordance with this model, CHS^−^ fruits contain lower levels of flavonoids than fruits of wild type *F. x ananassa* cv. Calypso (Lunkenbein et al., 2006), resulting in increased lignin content in CHS^−^ fruit upon agroinfiltration of different constructs in comparison with CHS^−^/pBI-intron control (Figure 10). Besides, auxin affects growth of strawberries and early fruit development and delays ripening (Given et al., 1988) whereas fruit ripening is associated with rapid softening (Zhang et al., 2011). Thus, we assumed that increased auxin transport in CHS^−^ fruits after agroinfiltration led to delayed ripening, which impacts on fruit firmness in addition of enhanced *FaPOD27* expression induced by infection with *Agrobacterium*.

Although lignin provides structural support for all land plants, until recently, only carbohydrate polymers such as pectin and cellulose and to a lesser extent proteins such as the expansins have been regarded as structure forming and stabilizing components in fruits. Microarray analyses, however, have provided evidence that the expression of specific genes involved in lignin formation affect strawberry fruit firmness. In this study, the analyses of *CCR*. *CAD*, and *POD* genes from *F. x ananassa* clearly revealed the significance of *FaPOD27* for lignin formation and firmness in strawberry fruit. The gene does not only function in late stages of strawberry fruit ripening but is also a component of the fruit's defense mechanism against bacterial invasion. The results can be used to develop strawberry varieties with improved firmness, storage stability, enhanced resistance, and thus fruit quality.

### Conflict of interest statement

The authors declare that the research was conducted in the absence of any commercial or financial relationships that could be construed as a potential conflict of interest.
