# Supplementary material for: FaPOD27 functions in the metabolism of polyphenols in strawberry fruit (Fragaria sp.)
Source: Front Plant Sci. 2014 Oct 9;5:518. doi: 10.3389/fpls.2014.00518 (PMC4191155; doi:10.3389/fpls.2014.00518)
Supplement: Supplementary file 1 [file DataSheet1.DOCX]

***Supplementary Material***

**FaPOD27 functions in the polyphenol metabolism of strawberry fruit (*Fragaria* sp.)**

Su-Ying Yeh, Fong-Chin Huang, Thomas Hoffmann, Mechthild Mayershofer and Wilfried Schwab*

Biotechnology of Natural Products, Technische Universität München, Liesel-Beckmann-Str. 1, D-85354 Freising, Germany

**Table S1**. Primers used for cloning of cDNAs and plasmid construction.

**Table S2**. Primers used for qRT-PCR analysis.

**Fig. S1** Trimethylsilylated thioacidolysis products from strawberry fruits infiltrated with different constructs detected by GC-MS analysis.

**Fig. S2** Alignment of amino acid sequences of CCR proteins from selected plants.

**Fig. S3** Expression of FaCCR protein in *E. coli* strain BL21(DE3)pLysS.

**Fig. S4** Effects of pH (A) and temperature (B) on recombinant GST-FaCCR activity.

**Fig. S5** LC-UV-ESI-MS^n^ analysis of the GST-FaCCR reaction containing mixture substrates.

**Fig. S6** Identification of products formed by recombinant GST-FaCCR.

**Fig. S7** Alignment of amino acid sequences of SAD and CAD proteins from selected plants.

**Fig. S8** FaCAD activity expressed in *S. cerevisiae*.

**Fig. S9** LC-UV-ESI-MS^n^ analysis of strawberry peroxidase reaction products.

**Fig. S10** Relative expression profiles of monolignol biosynthesis genes of *F. x ananassa* cv. Elsanta in response to wounding.

**Fig. S11** Metabolite levels in *FaCCR-*, *FaCAD-*, *FaPOD*-downregulated and -upregulated *F. x ananassa* cv. Elsanta fruits.

**Fig. S12** Relative expression profiles of individual *FaCCR-*, *FaCAD-*, *FaPOD*-downregulation and
-upregulation as well as combinations in *F. x ananassa* cv. Calypso (CHS^-^).

**Fig. S13** Thioacidolysis products formed from *O*-4-linked ferulic acid and differently treated strawberry fruits.

**Supplementary data**

**Table S1** Primers used for cloning of cDNAs and plasmid construction

| **Primer** | **Sequence (5’ to 3’)** | | | | | **Target gene/Plasmid** | | | | | | | |
| --- | --- | --- | --- | --- | --- | --- | --- | --- | --- | --- | --- | --- | --- |
| **Cloning** |  | | | | |  | | | | | | | |
| CCR-FD | ATGCCTGYYGATVHYWSMTC | | | | | | | *FaCCR* | | | | | |
| CCR-RD | TTATTGRATYTTSAHRGASTC | | | | | | |  |  |  |  |  |  |
| CAD-FD | ATGKCTATCGAGCAAGAACAC | | | | | | | *FaCAD* | | | | | |
| CAD-RD | TTAAGASCTARCCTTCAGTGT | | | | | | |  |  |  |  |  |  |
| gPOD F | ATGGGTTCCAGAGCTCTCTTC | | | | | | | *FaPOD* | | | | | |
| gPOD R | CTAGTGGAGCTTGTTGGCCAC | | | | | | |  |  |  |  |  |  |
| **Construction in protein expressing vectors** | | | | | | | | | | | | |  |
| CCR F_BamHI | | | | | | CAGGGATCCATGCCTGTTGATAACTG | | | | | *FaCCR* |  |  |
| CCR R_SmaI | | | | | | CTGCCCGGGTTATTGGATTTTGAAG | | | | |  |  |  |
| POD F_BamHI | | | | | | TCTGGATCCATGGGTTCCAGAGCTC | | | | | *FaCAD* |  |  |
| POD R_SmaI | | | | | | TATCCCGGGCTAGTGGAGCTTGTTG | | | | |  |  |  |
| CAD_F | | | GCTGGATCCAACACAATGTCTATCGAGCAAG | | | | | | | | *FaPOD* |  |  |
| XbaI _R | | | GCATCTAGAGTTAGCAGCCGGATCTCA | | | | | | | |  |  |  |
| **Construction in intron-hairpin vectors** | | | | | | | | | | | | |  |
| CCR-NheI F | | | CTAGCTAGCGTCGTCTTCACGTCTTC | | | | *FaCCR* | | | | |  |  |
| CCR-SpeI R | | | GCGACTAGTAGAATTGGCATAAGTCTT | | | |  |  |  |  |  |  |  |
| CAD-NheI F | | | CGCGCTAGCTTCTACAGAAGGGAAAC | | | | *FaCAD* | | | | |  |  |
| CAD-SpeI R | | | GCAACTAGTGTAGTACTTGGCACCGTA | | | |  |  |  |  |  |  |  |
| POD-NheI F | | | CATGCTAGCCTCTACAAGCGCCACAAG | | | | *FaPOD* | | | | |  |  |
| POD-SpeI R | | | GCGACTAGTATCTCTCCTTCCAGTT | | | |  |  |  |  |  |  |  |
| **Construction in overexpression vectors** | | | | | | | | | | | | |  |
| CCR F_BamHI | | | CAGGGATCCATGCCTGTTGATAACTG | | | *FaCCR* | | | | | |  |  |
| CCR R_SmaI | | | CTGCCCGGGTTATTGGATTTTGAAG | | |  |  |  |  |  |  |  |  |
| CAD F_BamHI | | | CGTGGATCCATGTCTATCGAGCAAG | | | *FaCAD* | | | | | |  |  |
| CAD R_SmaI | | | TATCCCGGGTTAAGAGCTAGCCTTC | | |  |  |  |  |  |  |  |  |
| POD F_BamHI | | | TCTGGATCCATGGGTTCCAGAGCTC | | | *FaPOD* | | | | | | | |
| POD R_SmaI | | | TATCCCGGGCTAGTGGAGCTTGTTG | | |  |  |  |  |  |  |  |  |

FD: degenerate forward primer; RD: degenerate reverse primer; F: forward primer; R: reverse primer
 Underlined indicates H=A/T/C, K=G/T, M=A/C, R=G/A, S=G/C, V=G/A/C, W=A/T, Y=T/C
 Engineered restriction sites are double underlined.

**Table S2** Primers used for qRT-PCR analysis

| **Primer** | **Sequence (5’ to 3’)** | | | **Target genes** | | | | | | |  |  |  |  |
| --- | --- | --- | --- | --- | --- | --- | --- | --- | --- | --- | --- | --- | --- | --- |
| **Gene-specific primers** | | | | | |  | | | | | | |  |  |
| CCR-F | GAGAGGCTATAATGTGAGAGGAACCGTCAG | | | | *FaCCR* | | |  |  |  |  |  |  |  |
| CCR-R | GCGATGCAGTGTGGAAAACGCCATCACAGC | | | |  |  |  |  |  |  |  |  |  |  |
| CAD-F | CATGGTCAAGAATGAATGGGGCTTCTCTAC | | | | *FaCAD* | | |  |  |  |  |  |  |  |
| CAD-R | GCACCGTAAGTGAGTATCTGTTTGGGGCAG | | | |  | | | | |  |  |  |  |  |
| POD-F | GCTGAGATCCTTGAGGAGTACCTTCCTGAC | | | | *FaPOD* | | | |  |  |  |  |  |  |
| POD-R | TGTGAGGGACATGGTCTGGGTTCAGAGCTG | | | |  |  |  |  |  |  |  |  |  |  |
| POD27-F | | ATTTCCATGATTGCTTTGTCA | | | | *FaPOD27* (Ring *et al*., 2013) | |  |  |  |  |  |  |  |
| POD27-R | | CAACGGCTAAGATGTCAGAAC | | | |  |  |  |  |  |  |  |  |  |
| PAL-F | | | TTGAAGCTCATGTCTTCCAC | *FaPAL* (Almeida *et al*., 2007) | | | | | | | | | | |
| PAL-R | | | CAAGTTCTCCTCCAAATG |  |  |  |  |  |  |  |  |  |  |  |
| CHS-F | | | GCTGTCAAGGCCATTAAGGA | *FaCHS* (Almeida *et al*., 2007) | | | | | | | | | | |
| CHS-R | | | GAGCAAACAACGAGAACACG |  |  |  |  |  |  |  |  |  |  |  |
| **Interspacer primers** | | | | | | | | | | | | | |  |
| IS-F | | | ACCGTTGATTCGCACAATTGGTCATCG | | | 16S-23S interspacer region | | | | | | |  |  |
| IS-R | | | TACTGCGGGTCGGCAATCGGACG | | |  |  |  |  |  |  |  |  |  |

F: forward primer; R: reverse primer


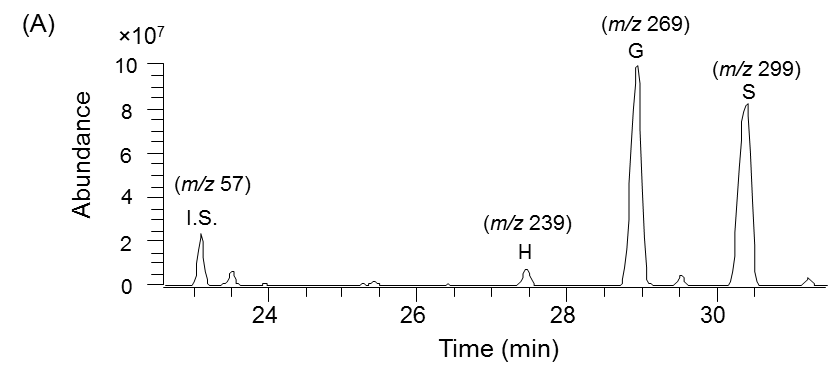

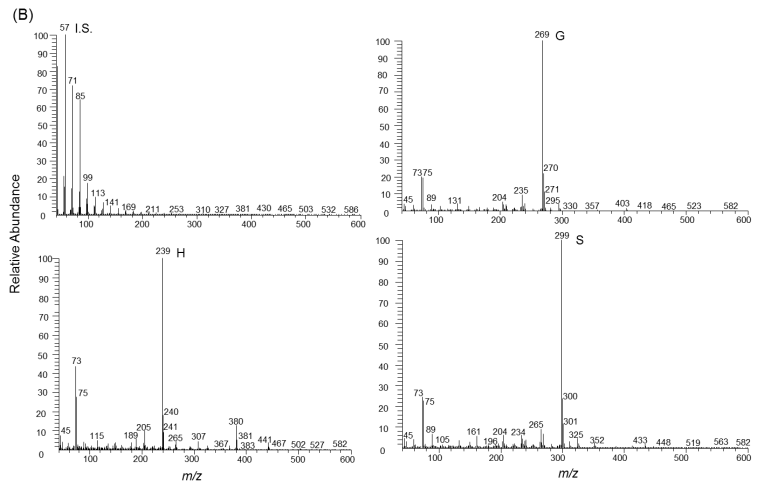


**Fig. S1** Trimethylsilylated thioacidolysis products from strawberry fruits infiltrated with different constructs detected by GC-MS analysis. (A) The ion chromatogram shows major monomeric products formed from H-, G-, and S-lignin, and the internal standard (I.S.). (B) Fragmentation pattern of the monomeric products and the internal standard. All fragments were confirmed by comparison with published data (Ralph *et al*., 2008; Palmer *et al*., 2008). *m/z* = mass-to-charge ratio.


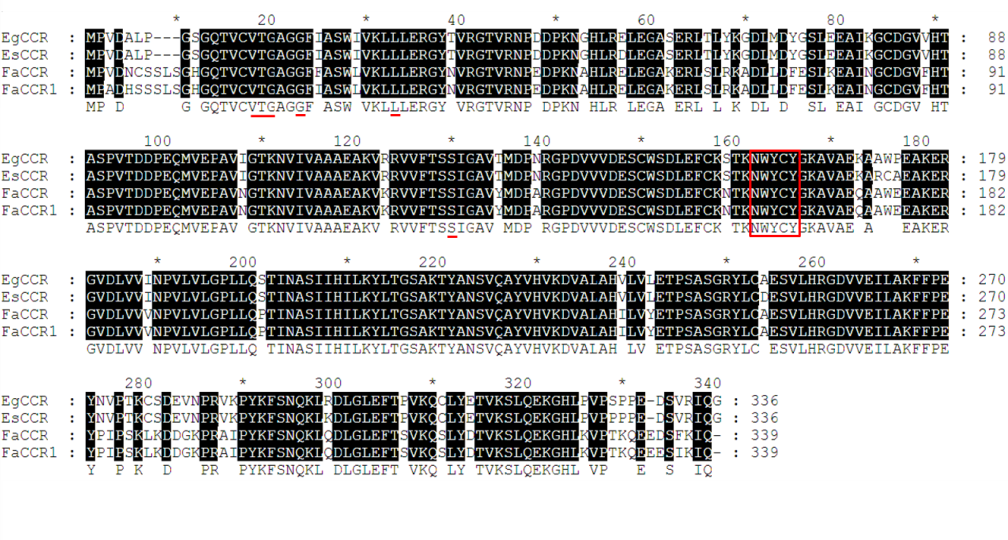


**Fig. S2** Alignment of amino acid sequences of CCR proteins from selected plants.

All sequences were aligned using the AlignX program of Vector NTI and GeneDoc software. A consensus sequence, found in a majority of the sequences, is shown below the sequence. Identical amino acids in the sequences are shaded in black. Red underlines indicate putative amino acids involved in cofactor binding. The putative signature of all CCRs is boxed. Protein sequences from other plants have been retrieved in the GenBank database and their accession numbers are as follows. EgCCR (*Eucalyptus gunnii*; X79566), EsCCR (*Eucalyptus* *saligna*; AF297877), FaCCR in this study (*F.* × *ananassa* cv. Elsanta), FaCCR1 (*F.* × *ananassa* cv. Elsanta, CCR-1 allele, accession AY285922).


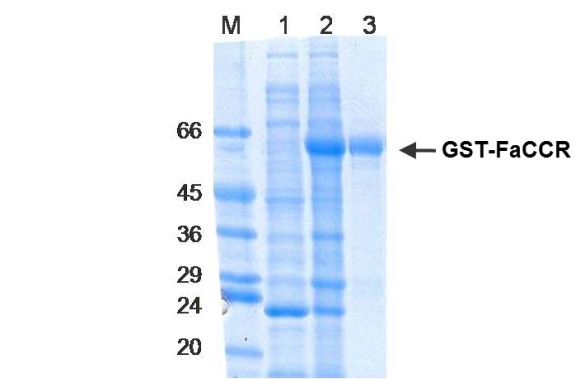


**Fig. S3** Expression of FaCCR protein in *E. coli* strain BL21(DE3)pLysS.

Lane (M) molecular mass markers; total protein extract of *E. coli* harboring *FaCCR* without IPTG (20 μg protein, lane 1) and after induction with IPTG (20 μg protein, lane 2); purified recombinant GST-FaCCR (5 μg protein, lane 3).


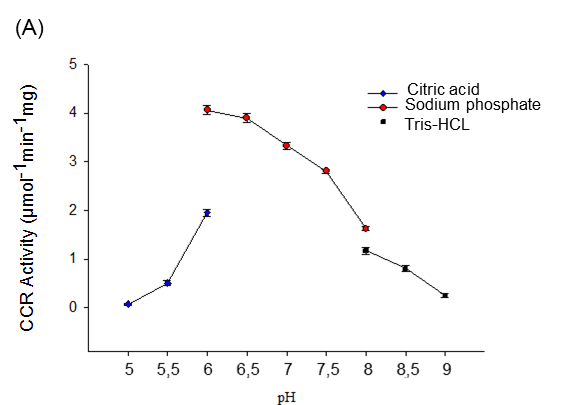

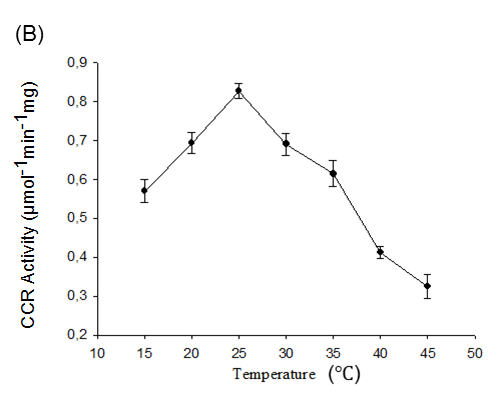


**Fig. S4** Effects of pH (A) and temperature (B) on recombinant GST-FaCCR activity.

(A) GST-FaCCR activity at various pH values and buffers was measured spectrophotometrically at 366 nm. (B) All reaction mixtures were incubated at different temperatures for 10 min and activity was determined by LC-UV-ESI-MS^n^. Each value is the mean of three independent samples and vertical bars represent standard errors.


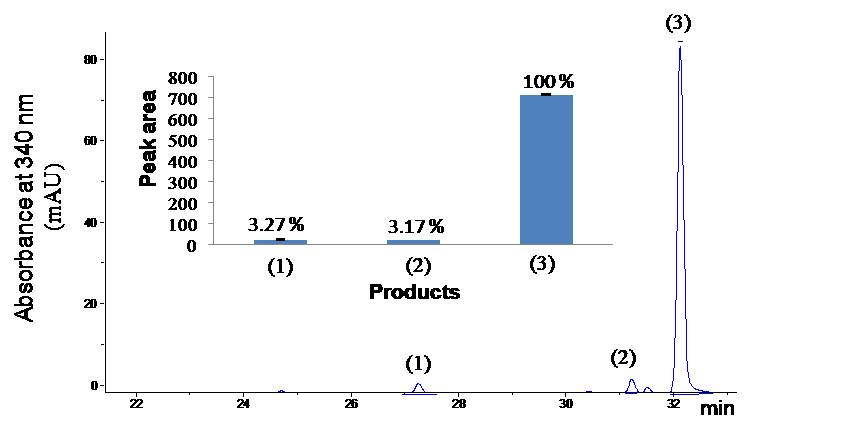


**Fig. S5** LC-UV-ESI-MS^n^ analysis of the GST-FaCCR reaction containing a mixture of equal molar concentrations of the substrates caffeoyl-CoA, *p*-coumaroyl-CoA, and feruloyl-CoA. Three products were monitored at 340 nm and were identified as (1) caffeic aldehyde, (2) *p*-coumaraldehyde, and (3) coniferaldehyde, respectively. Since molar extinction coefficients (ε340) of the products are almost identical (18.5 × 10^3^ M^-1^ cm^-1^ and 23.5 × 10^3^ M^-1^ cm^-1^ for coniferaldehyde and *p*-coumaraldehyde, respectively) they were not taken into account for the calculation of the relative enzyme activities (Somssich *et al*., 1996).


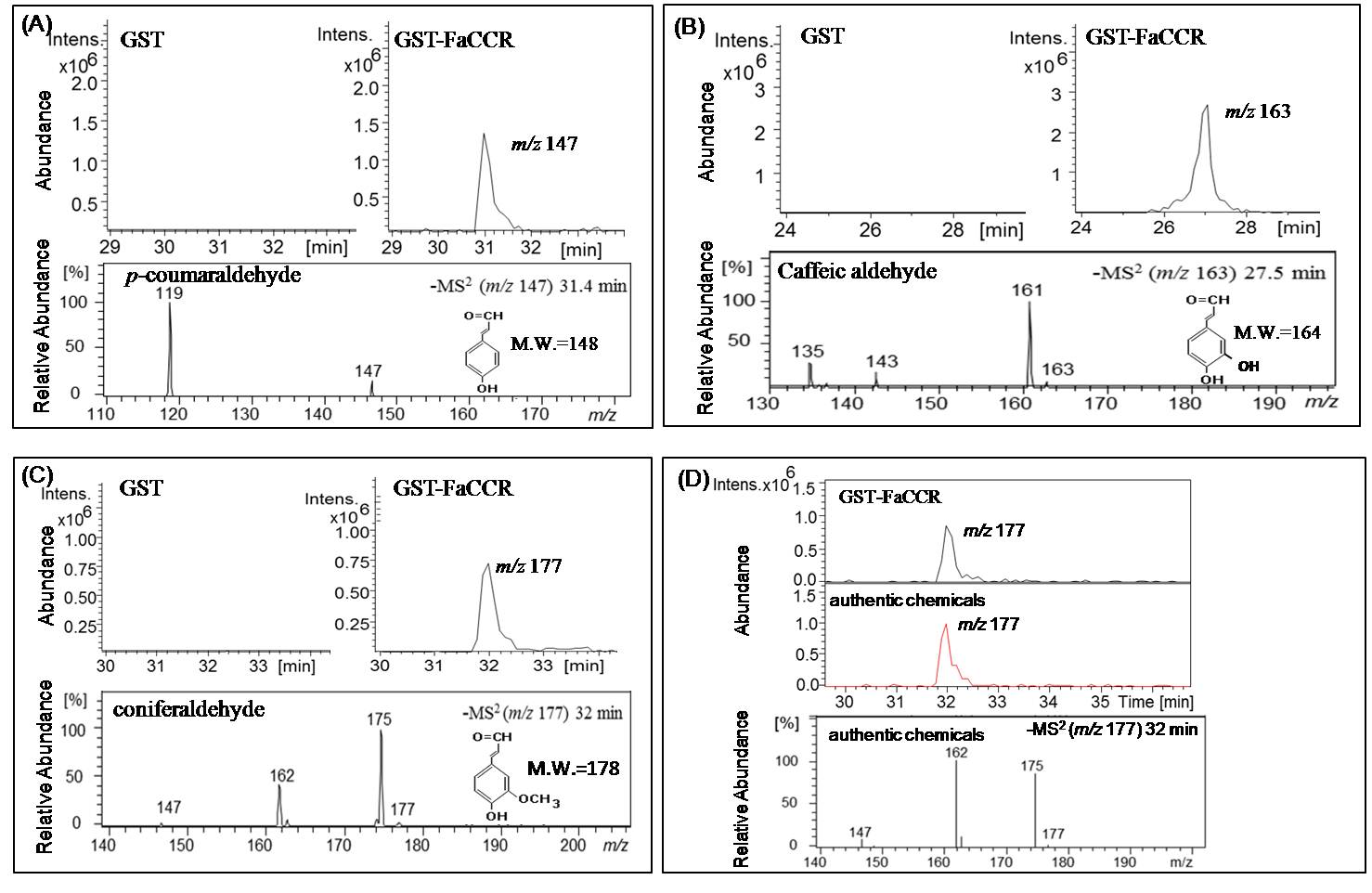


**Fig. S6** Identification of products formed by recombinant GST-FaCCR.

Reaction products and authentic coniferaldehyde (reference) were analyzed by LC-UV-ESI-MS^n^ and recorded at 340 nm (A-D_top). The MS^2^(-) spectra are showed (A-D_bottom). A GST-FaCCR reaction, with respective *p*-coumaroyl-CoA, caffeoyl-CoA, and feruloyl-CoA, was subjected to LC-UV-ESI-MS^n^. A single peak was generated that corresponded to its products: *p*-coumaraldehyde (A), caffeic aldehyde (B), and coniferaldehyde (C). No peaks were found in a GST (without GST-FaCCR proteins) reaction (A-C). MS spectral data for these three products was acquired in negative ion mode that targeted the [M-H]^-^ ions, which were observed at *m/z* 147, 163, and 177, respectively. Retention times and mass fragmentation patterns of the coniferaldehyde from GST-FaCCR products were identical to those from the authentic coniferaldehyde (D). M.W. molecular weight; *m/z* mass-to-charge ratio.


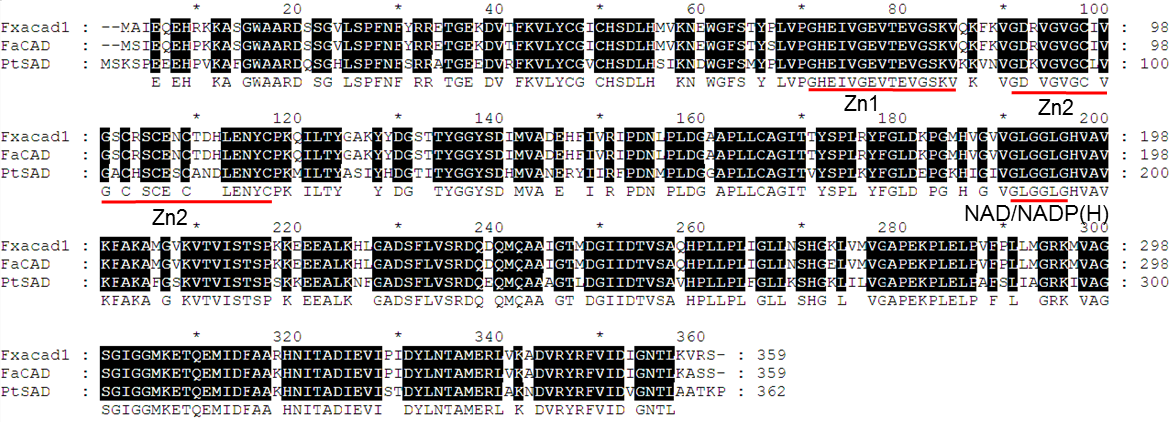


**Fig. S7** Alignment of amino acid sequences of SAD and CAD proteins from selected plants.

All sequences were aligned as described in Figure S2. Identical amino acids in the sequences are shaded in black. The red underline indicates putative amino acids involved in Zn1, Zn2, and NAD/NADP(H) binding motifs. Accession numbers are as follows: Fxacad1 (*F.* × *ananassa* cv. Chandler, U63534), FaCAD in this study (*F.* × *ananassa* cv. Elsanta), PtSAD (*Populus tremuloides*; AF273256).


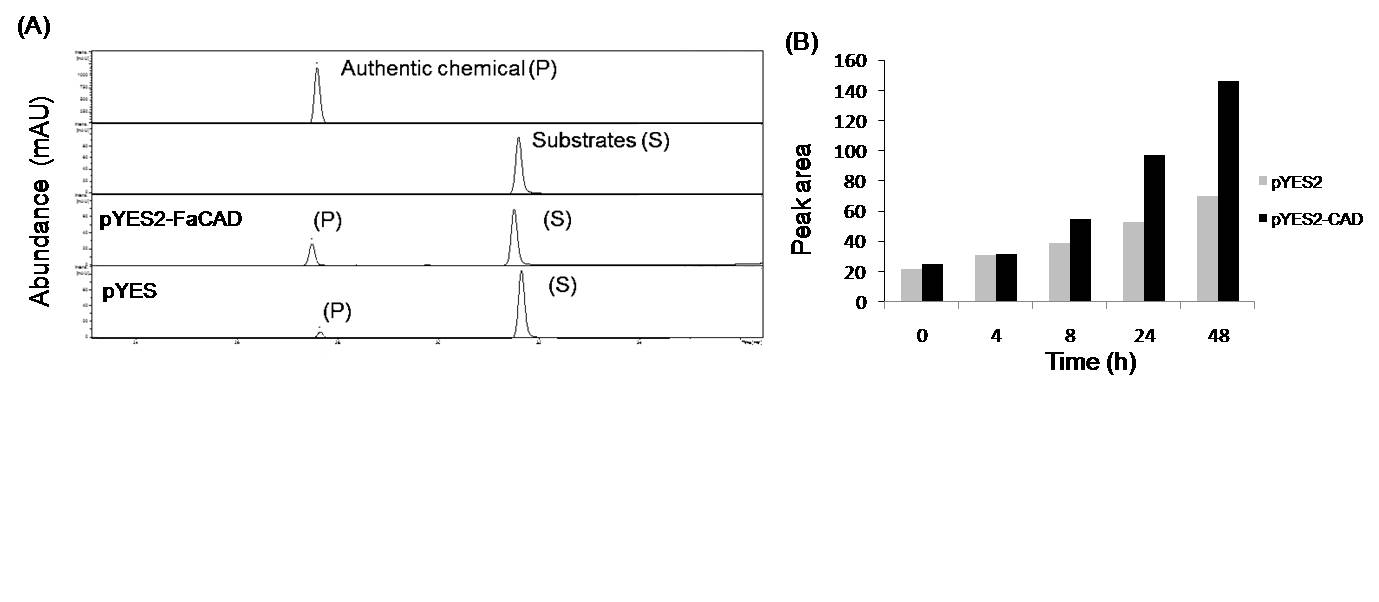


**Fig. S8** FaCAD activity expressed in *S. cerevisiae*.

Crude protein of pYES2 (control) and pYES2-FaCAD was used for enzyme activity assays. (A) Reaction products were identified by LC-UV-ESI-MS^n^ (S=coniferaldehyde, P=coniferyl alcohol). UV detection was done at 260 nm. (B) Coniferyl alcohol (product) formed in the pYES2 and pYES2-FaCAD reactions was quantified based on the peak areas of the UV traces.


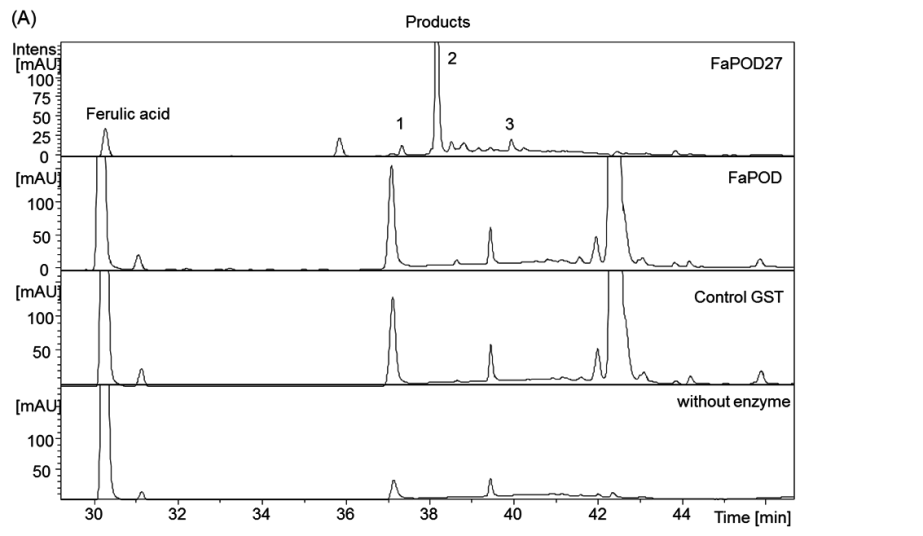

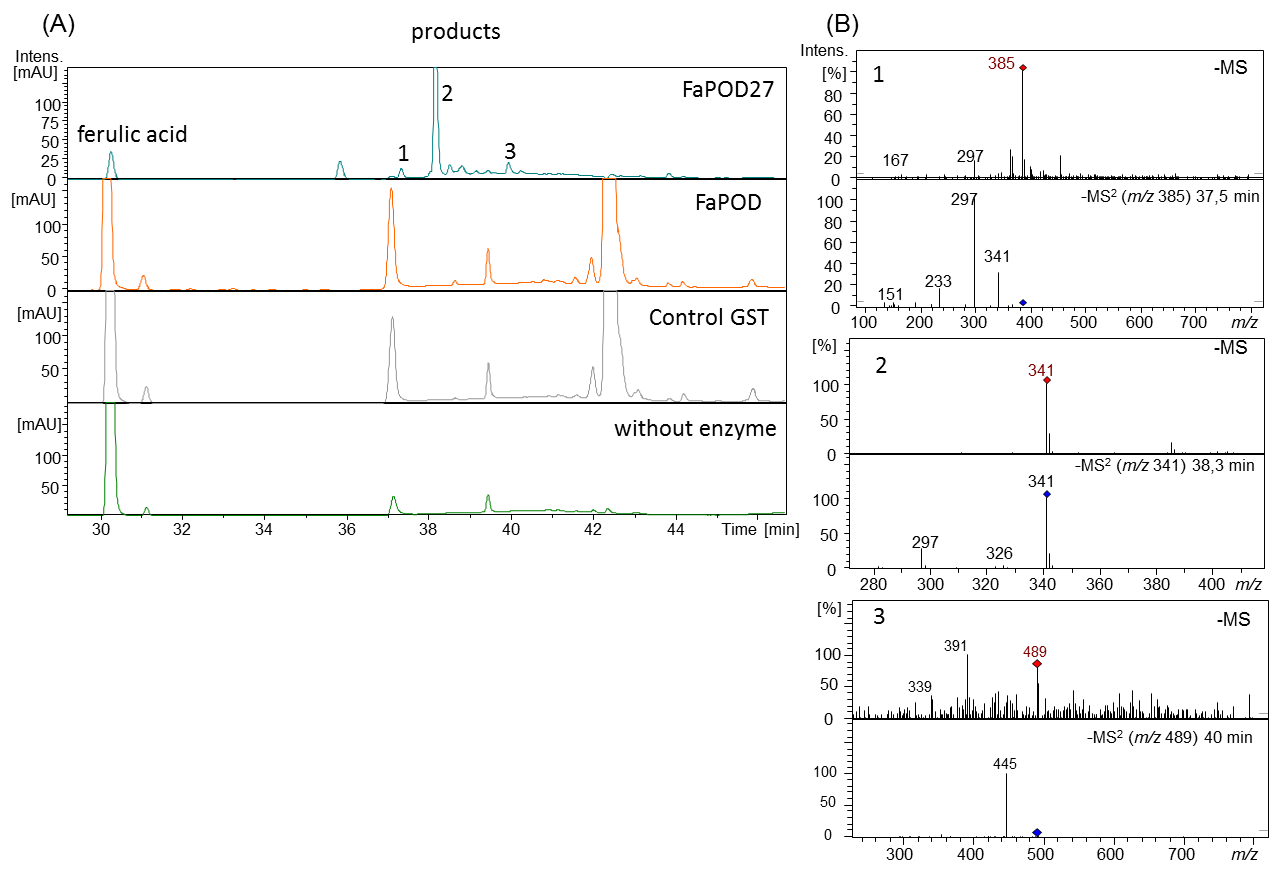


**Fig. S9** LC-UV-ESI-MS^n^ analysis of strawberry peroxidase reaction products.

(A) The reaction mixture contained the substrate ferulic acid and H_2_O_2_ with crude protein FaPOD27, FaPOD, control GST (pGEX-4X-1 vectors), or without enzymes. Substrate and products were monitored at 320 nm. The three products (A1-3) formed by FaPOD27 corresponded to the dehydrodimer of ferulic acid (B1), a decarboxylation product of the dehydrodimer precursor (B2), and unknown products (B3), respectively (Ward *et al*., 2001).


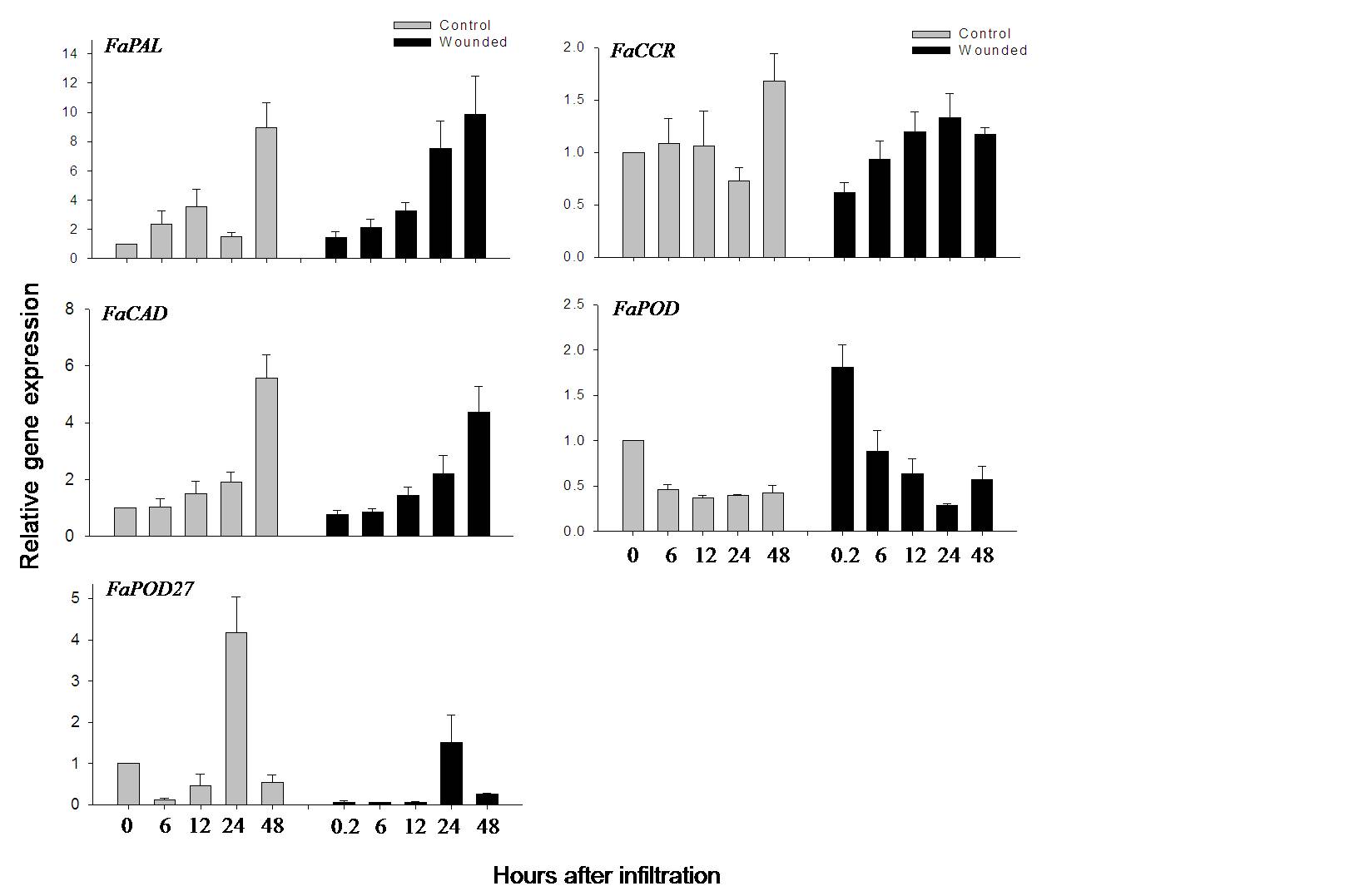


**Fig. S10** Relative expression profiles of monolignol biosynthesis genes of *F.* × *ananassa* cv. Elsanta in response to wounding.

Wounded fruits were harvested at 0.2 (12 min), 6, 12, 24 and 48 hr. Wild-type (untreated) fruits were used as controls and harvested at the same time points. Expression levels in the control (grey column) and wounded fruits (black column) were monitored by qRT-PCR using specific primers for the target genes (*FaPAL*, *FaCCR*, *FaCAD*, *FaPOD*, and *FaPOD*27) and an interspacer gene. The control fruit (0 h) was used as reference with one for each graph. For *FaCCR*, *FaCAD*, and *FaPOD*, values are mean ± SE of 9 triplicates from three independent fruits. For *FaPAL* and *FaPOD27*, values are mean ± SE of 4-6 replicates from two independent fruits. All values are


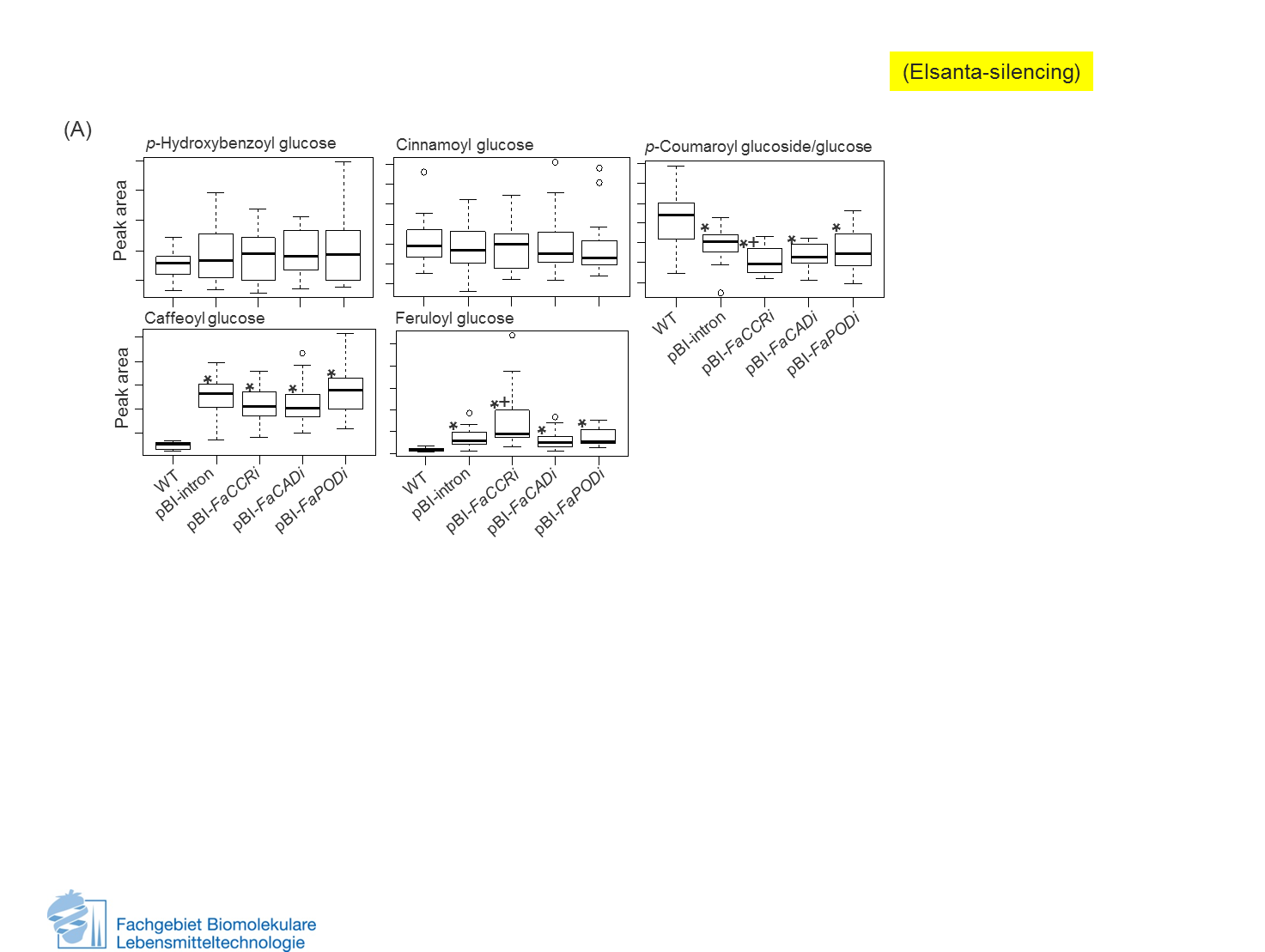


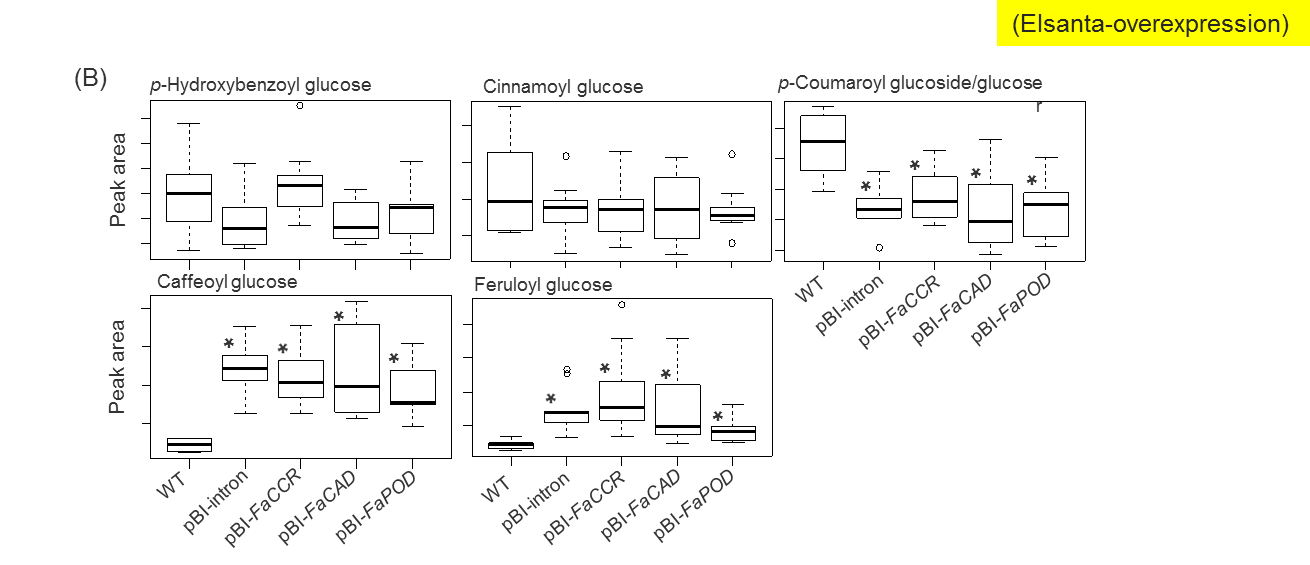


**Fig. S11** Metabolite levels in *FaCCR-*, *FaCAD-*, *FaPOD*-downregulated (A) and -upregulated *F.* × *ananassa* cv. Elsanta fruits (B).

(A) WT (Wild type; *n* =13), agroinfiltrated fruits with a non-ihpRNA construct (pBI-intron; *n*=20), and ihpRNA-mediated gene silencing constructs (pBI-*FaCCRi*; *n* =22, pBI-*FaCADi*; *n* =23, pBI-*FaPODi*; *n* =20). (B) WT (*n*=10), pBI-intron (*n*=10) and overexpression constructs (pBI-*FaCCR*; *n*=10, pBI-*FaCAD*; *n*=10, pBI-*FaPOD*; *n* =9). Wilcoxon-Mann-Whitney *U*-test was used for a non-parametric comparison of two groups. One asterisk (*) or a plus (+) in the box marks statistically significant decreased or increased levels (*P*<0.01) in comparison with WT and another group (indicated by*), or in comparison with pBI-intron and another group (indicated by+).

The following metabolites have been analyzed by LC-MS:

Phenolic acid derivatives:

*p*-hydroxybenzoyl glucose

cinnamoyl glucose

*p*-coumaroyl glucoside/glucose

caffeoyl glucose

feruloyl glucose

flavonoids

kaempferol glucoside

quercetin glucoside

catechin

anthocyanins

pelargonidin 3-glucoside

pelargonidin 3-glucoside-malonate

pelargonidin 3-rutinoside

proanthocyanidins

(epi)catechin-(epi)catechin isomer I

(epi)catechin-(epi)catechin isomer II

(Epi)afzelechin-(epi)catechin isomer I

(Epi)afzelechin-(epi)catechin isomer II


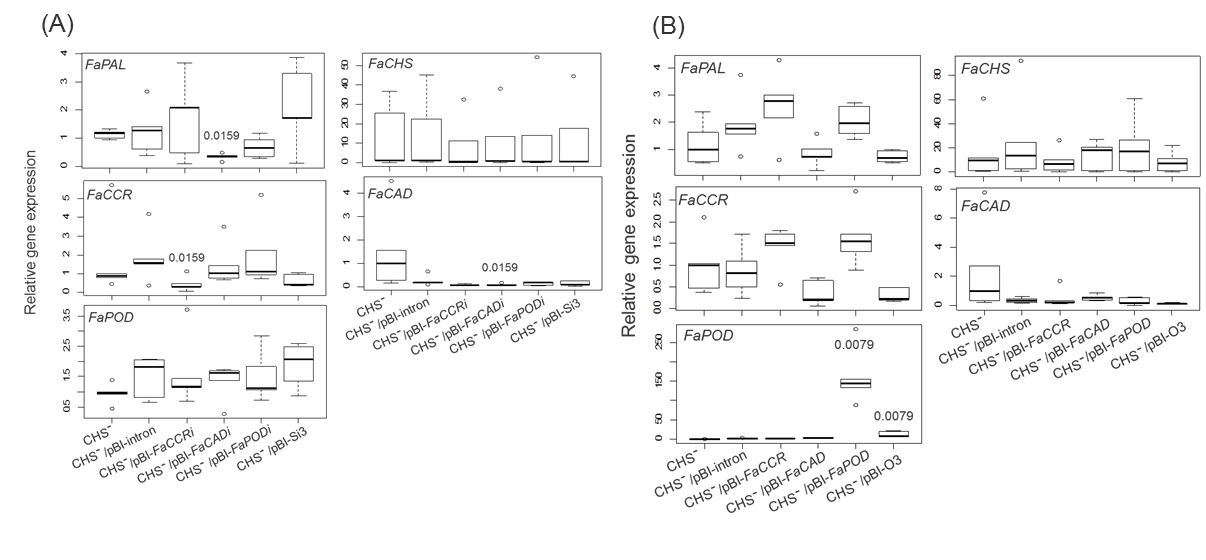


**Fig. S12** Relative expression profiles of individual *FaCCR-*, *FaCAD-*, *FaPOD*-downregulation (A) and upregulation (B) as well as combinations in *F.* × *ananassa* cv. Calypso (CHS^-^).

(A) Untreated Calypso fruits (CHS^-^), control fruits (CHS^-^/pBI-intron), down-regulated fruits (CHS^-^/pBI-*FaCCRi*, CHS^-^/pBI-*FaCADi*, and CHS^-^/pBI-*FaPODi*), and combined three genes (CHS^-^/pBI-Si3). (B) Up-regulated fruits (CHS^-^/pBI-*FaCCR*, CHS^-^/pBI-*FaCAD*, and CHS^-^/pBI-*FaPOD*), and combined three genes (pBI-O3). Expression levels of all samples were monitored by qRT-PCR. *FaPAL*, *FaCHS*, *FaCCR*, *FaCAD*, and *FaPOD* were target genes. For each box-plot graph, one of the CHS^-^ groups was used as the reference (set to one) and each group had five biological replicates. The Wilcoxon-Mann-Whitney *U-*test was used for a non-parametric comparison of two groups from CHS^-^/pBI-intron and another group. Values indicate statistically significant reduced or increased levels (*P* <0.02) and are shown in the box.


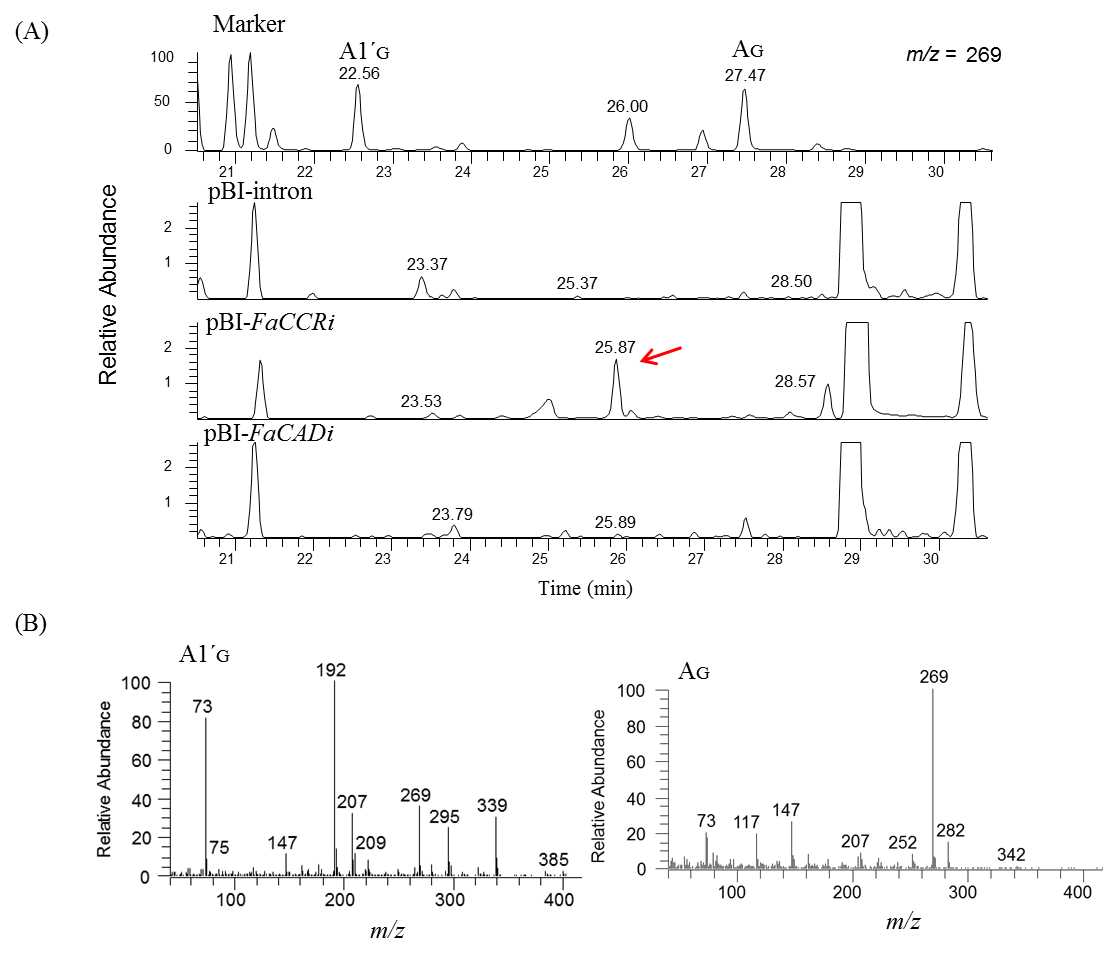


**Fig. S13** Thioacidolysis products formed from *O*-4-linked ferulic acid and differently treated strawberry fruits. (A) The ion chromatograms at *m/z* 269 show the ferulic acid markers A1´G (ferulic acid+EtSH) and AG (ferulic acid) at retention times 22.56 and 27.47 min, respectively and compounds obtained from pBI-intron, pBI-*FaCCRi*, and pBI-*FaCADi* treated fruit. (B) The identity of A1´G (ferulic acid+EtSH) and AG (ferulic acid) was confirmed by comparison of their mass spectra with published data (Ralph *et al*., 2008). The arrow indicates a new compound from pBI-*FaCCRi* treatment. *m/z* mass-to-charge ratio.
